# Phenome-wide analyses identify an association between the parent-of-origin effects dependent methylome and the rate of aging in humans

**DOI:** 10.1186/s13059-023-02953-6

**Published:** 2023-05-15

**Authors:** Chenhao Gao, Carmen Amador, Rosie M. Walker, Archie Campbell, Rebecca A. Madden, Mark J. Adams, Xiaomeng Bai, Ying Liu, Miaoxin Li, Caroline Hayward, David J. Porteous, Xueyi Shen, Kathryn L. Evans, Chris S. Haley, Andrew M. McIntosh, Pau Navarro, Yanni Zeng

**Affiliations:** 1 Faculty of Forensic Medicine, Zhongshan School of Medicine, Sun Yat-Sen University, Guangzhou, China; 2 MRC Human Genetics Unit, Institute of Genetics and Cancer, University of Edinburgh, Edinburgh, UK; 3 Centre for Clinical Brain Sciences, Chancellor’s Building, 49 Little France Crescent, Edinburgh BioQuarter, Edinburgh, UK; 4 Centre for Genomic and Experimental Medicine, Institute of Genetics and Cancer, University of Edinburgh, Edinburgh, UK; 5 School of Psychology, University of Exeter, Perry Road, Exeter, UK; 6 Division of Psychiatry, University of Edinburgh, Edinburgh, UK; 7 Zhongshan School of Medicine, Sun Yat-Sen University, Guangzhou, China; 8 Roslin Institute and Royal (Dick) School of Veterinary Studies, University of Edinburgh, Edinburgh, UK; 9 Guangdong Province Translational Forensic Medicine Engineering Technology Research Center, Zhongshan School of Medicine, Sun Yat-Sen University, Guangzhou, China; 10 Guangdong Province Key Laboratory of Brain Function and Disease, Zhongshan School of Medicine, Sun Yat-Sen University, Guangzhou, China

**Keywords:** Atypical parent-of-origin effect, Aging, DNAmTL acceleration, Maternal smoking exposure, Lifestyle, Intelligence

## Abstract

**Background:**

The variation in the rate at which humans age may be rooted in early events acting through the genomic regions that are influenced by such events and subsequently are related to health phenotypes in later life. The parent-of-origin-effect (POE)-regulated methylome includes regions enriched for genetically controlled imprinting effects (the typical type of POE) and regions influenced by environmental effects associated with parents (the atypical POE). This part of the methylome is heavily influenced by early events, making it a potential route connecting early exposures, the epigenome, and aging. We aim to test the association of POE-CpGs with early and later exposures and subsequently with health-related phenotypes and adult aging.

**Results:**

We perform a phenome-wide association analysis for the POE-influenced methylome using GS:SFHS (*N*_discovery_ = 5087, *N*_replication_ = 4450). We identify and replicate 92 POE-CpG-phenotype associations. Most of the associations are contributed by the POE-CpGs belonging to the atypical class where the most strongly enriched associations are with aging (DNAmTL acceleration), intelligence, and parental (maternal) smoking exposure phenotypes. A proportion of the atypical POE-CpGs form co-methylation networks (modules) which are associated with these phenotypes, with one of the aging-associated modules displaying increased within-module methylation connectivity with age. The atypical POE-CpGs also display high levels of methylation heterogeneity, fast information loss with age, and a strong correlation with CpGs contained within epigenetic clocks.

**Conclusions:**

These results identify the association between the atypical POE-influenced methylome and aging and provide new evidence for the “early development of origin” hypothesis for aging in humans.

**Supplementary Information:**

The online version contains supplementary material available at 10.1186/s13059-023-02953-6.

## Background

Aging is a multi-system process manifesting as a progressive decline of physiological integrity, impaired functions, and increased risk of adult-onset diseases and death [1]. Although everyone ages chronologically, the actual biological state, namely biological age, varies even among individuals of the same chronological age [2, 3]. The increased or delayed biological aging after accounting for chronological age has been defined as “age acceleration,” which can be estimated by biomarkers such as DNA methylation [4–7]. The identification of risk factors and biomarkers is crucial for the understanding of aging [2]. Genetic studies have reported large numbers of genomic loci associated with biological aging [8]. The proportion of biological aging explained by the heritable DNA sequence variation, however, only accounts for the influences from predisposing and unchangeable risk factors. Environment-involved effects such as epigenetic changes in response to life events, on the other hand, are flexible and reversible, representing a different collection of factors which could potentially better explain the dynamic nature of aging process across the lifespan.

Among all the environmental factors, early and developmental exposures are of particular interest. In 1994, Barker proposed a hypothesis that late-onset diseases can be profoundly influenced by early-life experiences [9]. Since then, a number of studies have provided evidence for the “early development of origin” hypothesis for adult-onset diseases such as schizophrenia and dementia [10, 11]. Aging, which is the biggest risk factor for many late-onset diseases, has been found to be associated with the environmental factors individuals are exposed at adulthood such as smoke and sun [12, 13]. When it comes to early effects, a few studies reported the associations of early exposures such as prenatal air pollution and early developmental event such as trisomy 21 with age acceleration in newborns and children [14, 15]. Whether those associations persist into adulthood is something that has not been widely studied. Therefore, the connections between early events/exposures and adulthood aging, and the molecular paths mediating any such connections, have been largely unexplored.

**Fig. 1:**
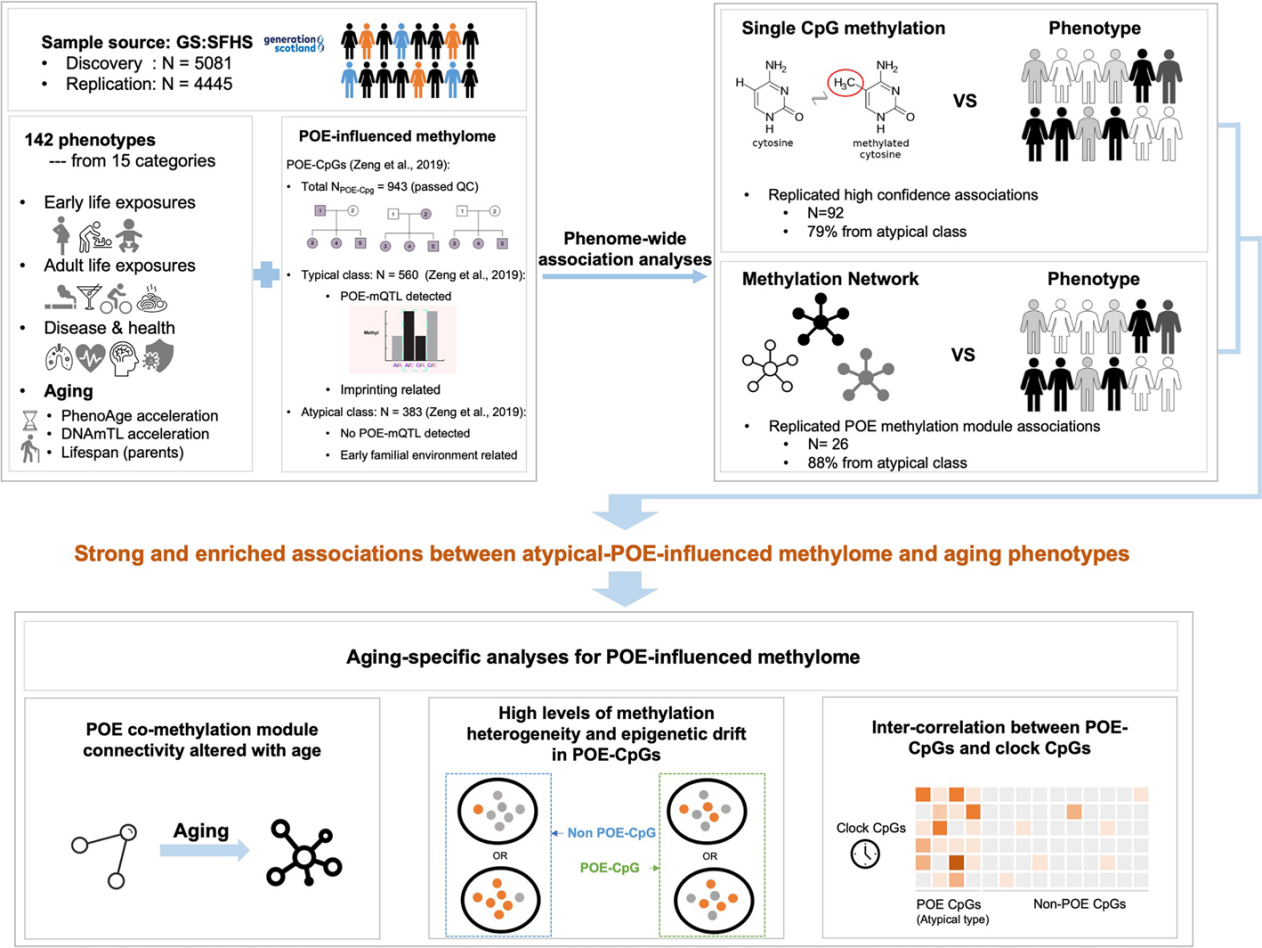
An overview of the study design. The illustrations of POE patterns are adapted from Zeng et al. [20, 25]

Parent-of-origin effects (POEs) are found in a subset of genomic regions that are highly sensitive to early-life events and associated with health outcomes at both early- and late-life stages [16–18]. For a DNA methylation site, the POE at the individual level was traditionally defined as any genetic effect of magnitude dependent on the parent-of-origin inheritance of alleles [19]. At the population level, we previously reported that the POE-influenced methylome manifested imbalanced methylation similarity between nuclear family members of the same genetic distance (mother–offspring, father-offspring, sibling-sibling pairs) (Fig. 1) [20]. The CpG sites displaying the imbalanced methylation pattern could be further divided into two groups, the typical and the atypical types (Fig. 1). The typical POE-CpGs are the group for which specific regulatory SNPs (POE-mQTLs) have been identified for influencing the methylation levels of the target CpGs through introducing the parent-of-origin-dependent SNP effect [17, 20]. This type of POE-CpGs is highly enriched in the regions targeted by genomic imprinting, a biological process happening at early developmental stages and the resulted epigenetic status needed to be well maintained/regulated throughout the life [17]. The epigenomic features influenced by the typical POEs have been found to be sensitive to prenatal and postnatal environmental stimuli, such as maternal nutrition during pregnancy and stress accompanied with assisted reproductive technologies [16, 21–24]. In contrast, some methylation sites also display the imbalanced methylation patterns but have no identified POE-mQTLs, and they are not enriched in known imprinted regions. These sites should, therefore, be regarded as “atypical POE-CpGs” [20]. Since dominance genetic effects have been ruled out for the majority of these atypical POE-CpGs [20], potential explanations for the atypical POE pattern are either small POE-mQTL (imprinting) effect not yet detected due to the lack of power, or early familial environmental effects introduced by the parents [20]. In any case, both typical and atypical POE-CpGs represent classes of CpGs for which methylation levels are heavily influenced by early-life events. If involved in the physiological functions in later life, they can be pivotal to the interplay between early-life experiences, epigenome, and adulthood health [16, 25].

The early-life-event-sensitive nature of the POE-regulated methylome renders it a plausible mechanism for the “early development of origin” hypothesis of adult aging. The link between POE and aging has been suggested by a few animal studies including one showing that the knockout of the imprinted gene *RasGrf1* promoted longevity [26], and further two showing that early-life adversity caused the deregulation of imprinting in the gene *Cdkn1c*, resulting in interrupted expression which influences aging-associated obesity [27, 28]. Human studies on the association between POE and aging, however, are very limited. When studying human samples, most research targeted rare developmental diseases (mainly imprinted disorders) caused by genetic mutation, others mainly examined the genetic effects that influence complex traits in a parent-of-origin way [29–31]. These included studies focused on late-onset diseases such as Alzheimer’s disease [32], but few have studied aging phenotypes (such as age acceleration) themselves. Moreover, even to examine aging phenotypes in future studies, the genotype-based strategies which the majority of existing human studies rely on do not account for the environment-sensitive and dynamic features of the POE-influenced genomic regions, which may lead to underestimation of the effects from these regions. Methylation studies, on the contrary, have the potential to capture the effects from both genetic background and environmental exposures, offering unique advantages in this context. To date, only one human study has reported the association between methylation levels of POE-influenced genes and the change of brain structures over time, but with a relatively small sample size (*N* = 485), and it only investigated a small proportion of POE-influenced genes (13 imprinted locations) [33]. Therefore, a well-powered systematic examination of the associations between the POE-regulated methylome and adult aging is warranted.

In this study, we aimed to investigate the POE-influenced methylome to collect evidence for the “early development of origin” hypothesis for aging in humans (Fig. 1). At both the single CpG and the co-methylation network levels, the associations between 943 POE-CpGs (*N*_typical_ = 560, *N*_atypical_ = 383) and 142 phenotypes were tested and replicated using two subsets of the Generation Scotland: Scottish Family Health Study (GS:SFHS. *N*_discovery_ = 5081, *N*_replication_ = 4445), a large family-based population cohort with genome-wide DNA methylation data (*N*_sites_ = 734,436), records of early- to late-life exposures and extensive health-related phenotypes available for participants [34, 35]. The phenotypes included four aging measurements: two epigenetic-based acceleration variables—DNAmTL acceleration and PhenoAge acceleration, and parental lifespans. As aging is the underlying cause of many adulthood illnesses, we expected widespread associations between aging-associated POE-CpGs with health-related phenotypes; therefore, a phenome-wide scan was applied instead of only testing for a few aging phenotypes. Our primary results revealed strongly enriched associations of the atypical POE-CpGs with early- and late-life exposures and with aging-related phenotypes at both the single CpG and co-methylation network levels. An aging-associated atypical POE co-methylation module whose internal methylation connectivity increased with age was further identified. These findings motivated two additional aging-focused analyses, which revealed high levels of methylation heterogeneity and epigenetic drift in the atypical POE-CpGs and intrinsic connections between the atypical POE-CpGs and clock CpGs (Fig. 1).

## Results

### Collection of POE-influenced methylation sites and their two subtypes

The list of 984 POE-CpGs was extracted from Zeng et al. [20]. Those CpGs displayed imbalanced methylation similarity between mother–offspring, father-offspring, and siblings (Fig. 1) and were identified using overlapping samples with the current discovery dataset [20]. Among them, 943 POE-CpGs passed the quality control for the DNA methylation data in this study. Below, we displayed the results from the analyses performed separately for the POE-CpGs belonging to the atypical and typical subgroups. The distinct features of these subgroups have been revealed previously: the typical POE-CpGs (*N*_QCed_typical_ = 560) are strongly enriched for imprinted regions and have POE-mQTLs detected, whereas the atypical POE-CpGs (*N*_QCed_typical_ = 383) are not enriched for imprinted regions and have no POE-mQTLs detected (Fig. 1) [20].

### Phenome-wide association analyses identified strong and enriched associations of the atypical POE-CpGs with aging, intelligence, and early/late environmental exposures

To identify the association between the methylation levels at each POE-CpG site and each phenotype, we applied the Mix-linear-model-based Omic Association (MOA) [36], a linear mixed model method that adjusts for the global correlation between probes, to account for unobserved confounders in the phenome-wide scan (*N*_CpG_ = 934, *N*_phenotype_ = 142) and the replication of significant results (“Methods”). For the replicated results, we additionally used the CpG outcome model, a classical linear regression model which avoids pre-adjustment of methylation-related variables to further validate the robustness of the MOA results (see “Methods” section).

At the discovery stage (*N*_sample_ = 5081), a total of 115 POE-CpG-phenotype pairs exceeded the phenome-wide significant threshold in the MOA analyses (FDR < 0.05 threshold: *P* ≤ 4.33 × 10^−5^). At the replication stage (*N*_sample_ = 4445), 85.2% of the POE-CpG-phenotype associations were statistically replicated at the FDR < 0.05 level (*N*_replicated_ = 98. Details in Additional file 2: Table S1). The CpG outcome model further validated the robustness of 94% (*N*_validated_ = 92 at FDR < 0.05) of the replicated associations reported by the MOA method (Additional file 2: Table S1), we considered this set as “high-confidence associations” (Additional file 2: Table S2).

**Fig. 2:**
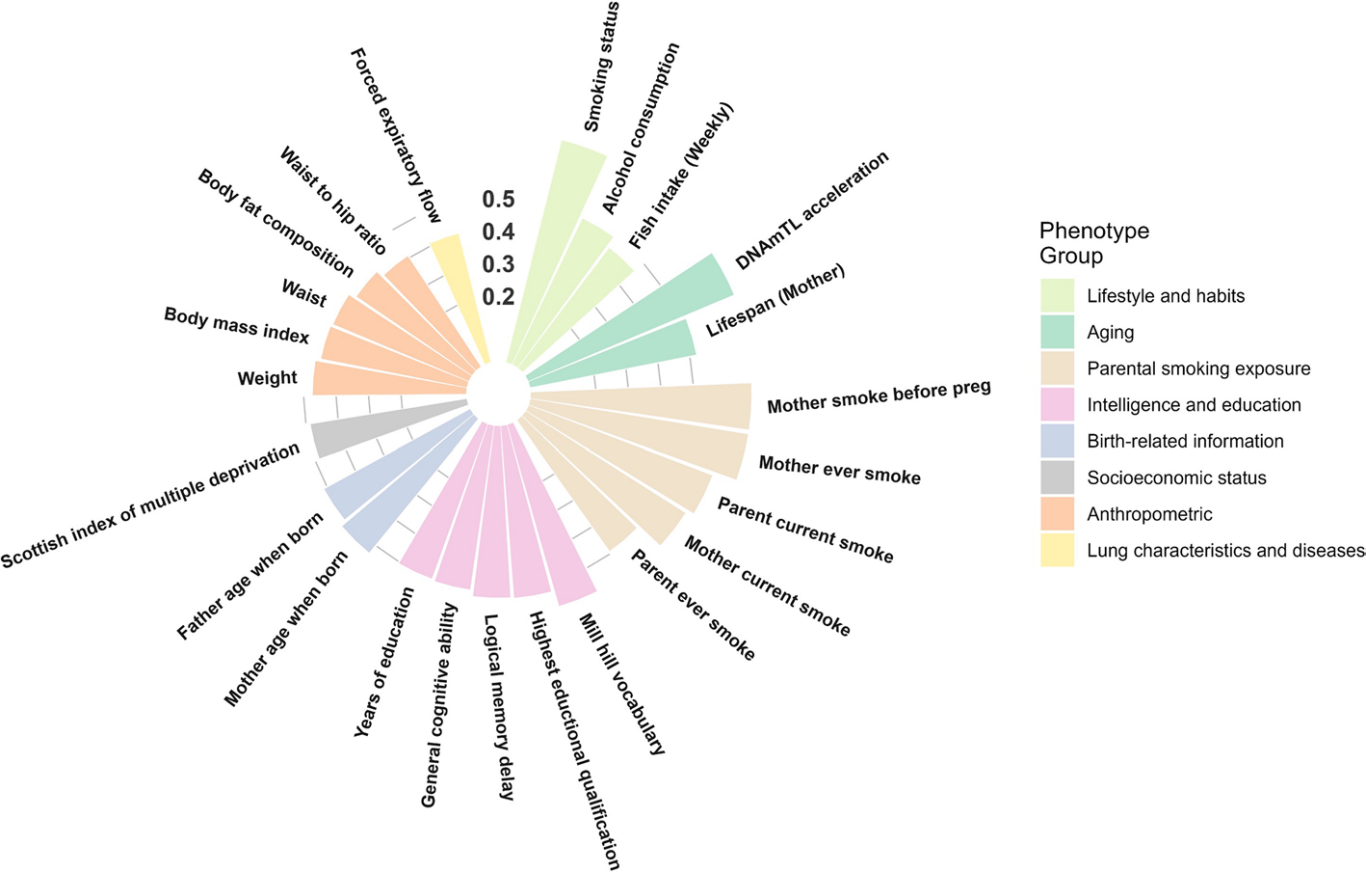
Significance level of the associations between phenotypes and POE-CpGs. Each bar represents a phenotype associated with at least one POE-CpG. The height of the bar represents the mean of the minus log-transformed *P* values of all POE-CpGs and the given phenotype

**Fig. 3:**
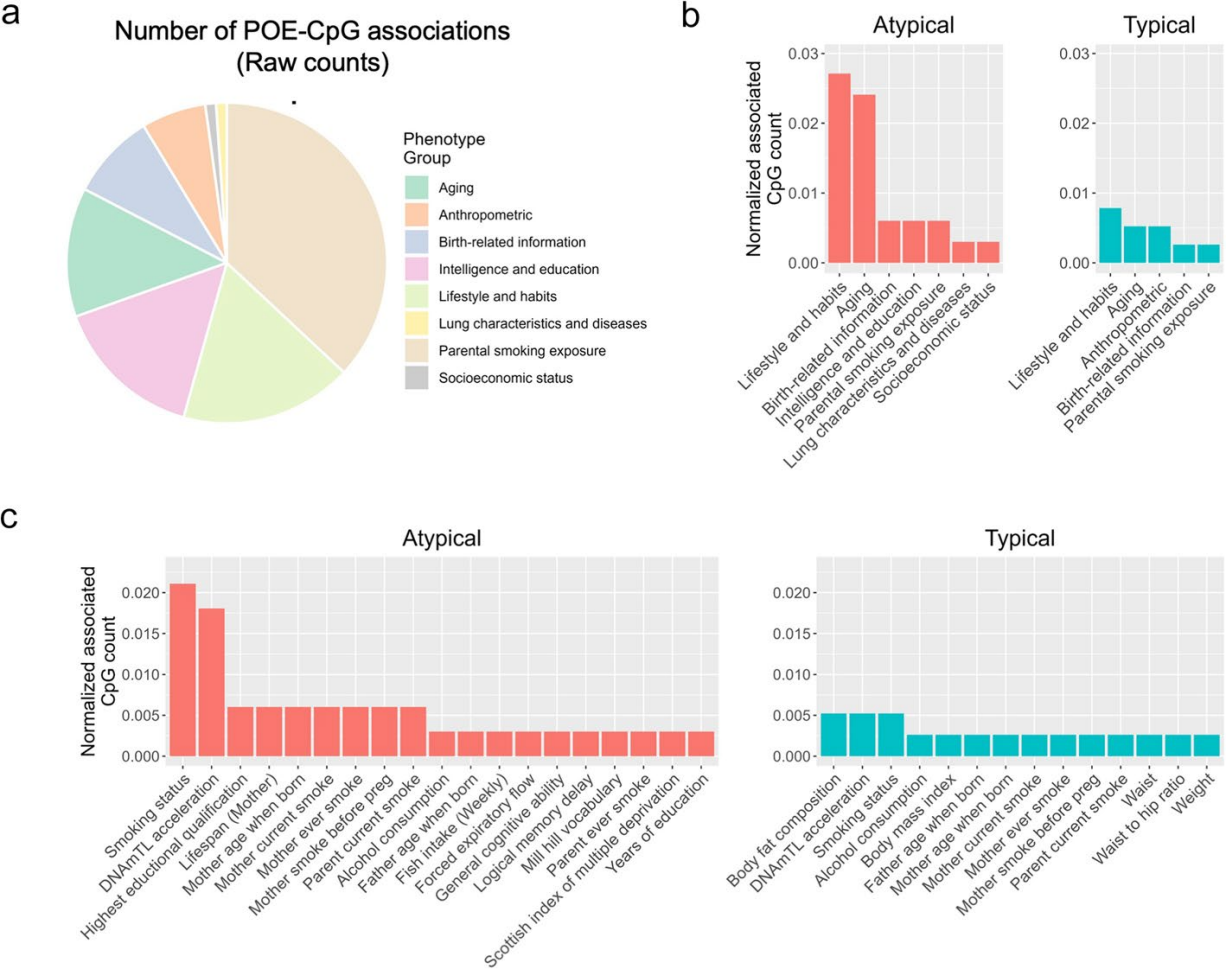
The raw and normalized count of the associated POE-CpGs per phenotype and per phenotypic category. **a** Raw counts. **b**,**c** Normalized counts. This was calculated by normalizing the raw counts by the total number of POE-CpGs in the atypical or typical group, and the correlation structures between POE-CpGs (multiple correlated CpGs were only counted as one). The number of the associated POE-CpGs was counted separately in the atypical and typical groups. **b** Normalized counts at categorical level. **c** Normalized counts at phenotypic level

**Fig. 4:**
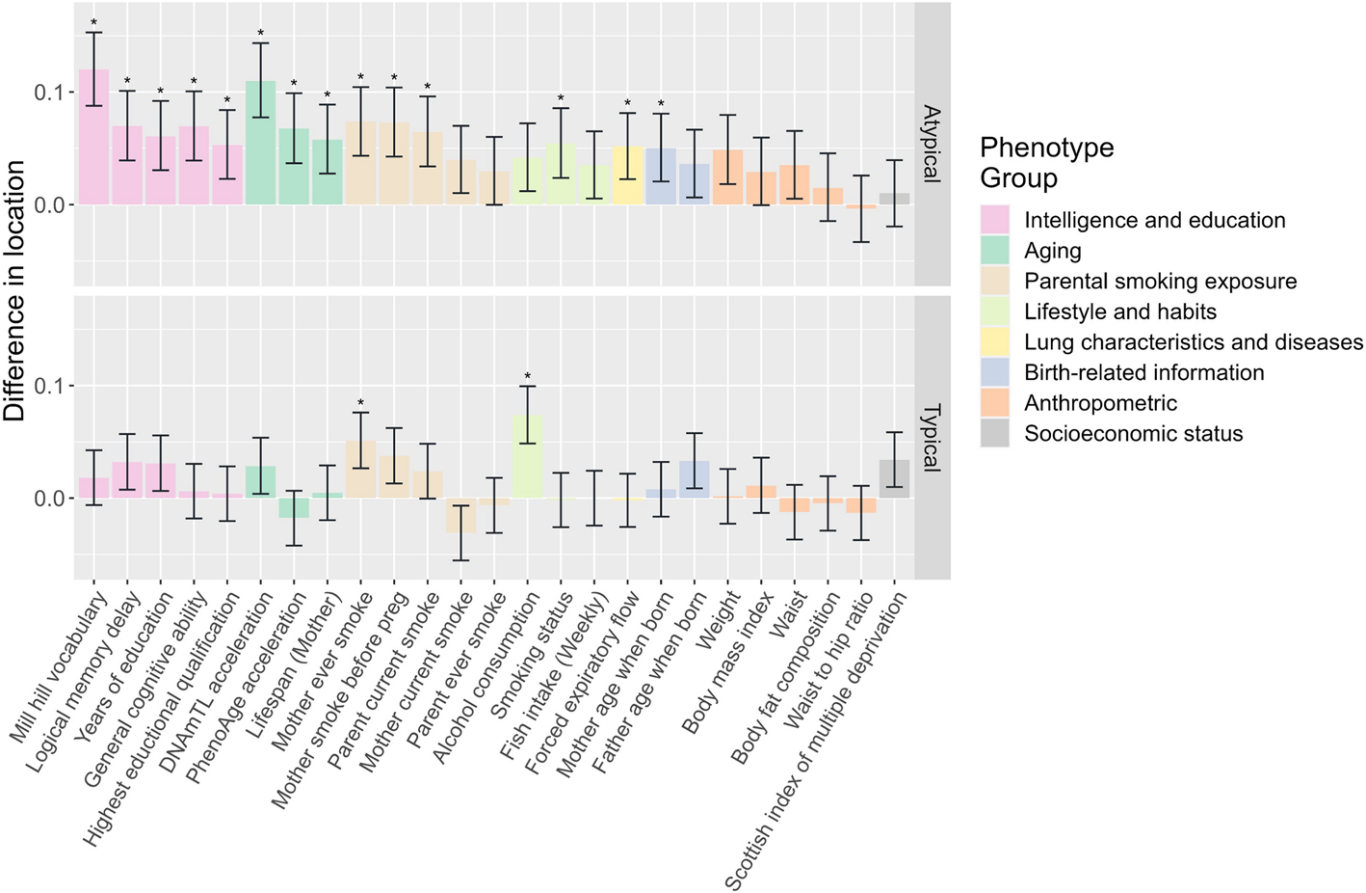
Wilcox test results for the comparisons between the association signals of the methylation sites from POE regions and that from non-POE regions. The analyses were performed separately for CpGs in the atypical and typical POE groups. Difference in location: the median of the difference between the associations from POE regions and the associations from non-POE regions, with positive values indicating stronger (enriched) associations from POE regions. “*”: the *P* value of the Wilcox test was significant after Bonferroni correction

The 92 high-confidence associations involved 38 POE-CpGs and 24 phenotypes, revealing widespread associations of POE-CpGs with multiple phenotype categories (Fig. 2). The atypical POE-CpGs contributed the majority of the associations (79.3%) (Additional file 2: Table S2), despite the fact that the atypical group only accounted for 40.6% of the total POE-CpGs. The phenotypic categories contributing the largest number of associations were parental smoking exposure, lifestyle, intelligence, and aging (Fig. 3a). Conditional analyses suggested that the associations with parental smoking were mainly driven by maternal smoking (Additional file 2: Table S3). We next ranked the phenotypic categories by the normalized counts of associated POE-CpGs after accounting for the baseline numbers of POE-CpGs in the atypical and the typical group respectively, as well as the correlation of methylation levels among POE-CpGs (Fig. 3b). This revealed that lifestyle and aging were the most associated phenotypic categories for POE-CpGs, in particular for the atypical group (Fig. 3b). Smoking status and DNAmTL acceleration were the most associated phenotypes (Fig. 3c). After annotating POE-CpGs onto functional regions, a significant enrichment was detected for the maternal-smoking-exposure–associated atypical POE-CpGs in CpG islands (Additional file 1: Fig. S1, Additional file 2: Table S4). In the comparisons of each phenotype’s association with POE-CpGs vs. the association with the rest of the methylome (non-POE-CpGs), a strong “atypical POE” enrichment was detected for multiple intelligence, aging, parental smoking exposure, and lifestyle phenotypes, with verbal intelligence (Mill Hill vocabulary test score) and DNAmTL acceleration displaying the strongest enrichment (Fig. 4, Additional file 2: Table S5); in contrast, for the “typical POE,” only weak enrichments were detected in a few phenotypes (alcohol consumption and maternal smoking exposure) (Fig. 4, Additional file 2: Table S5).

**Fig. 5:**
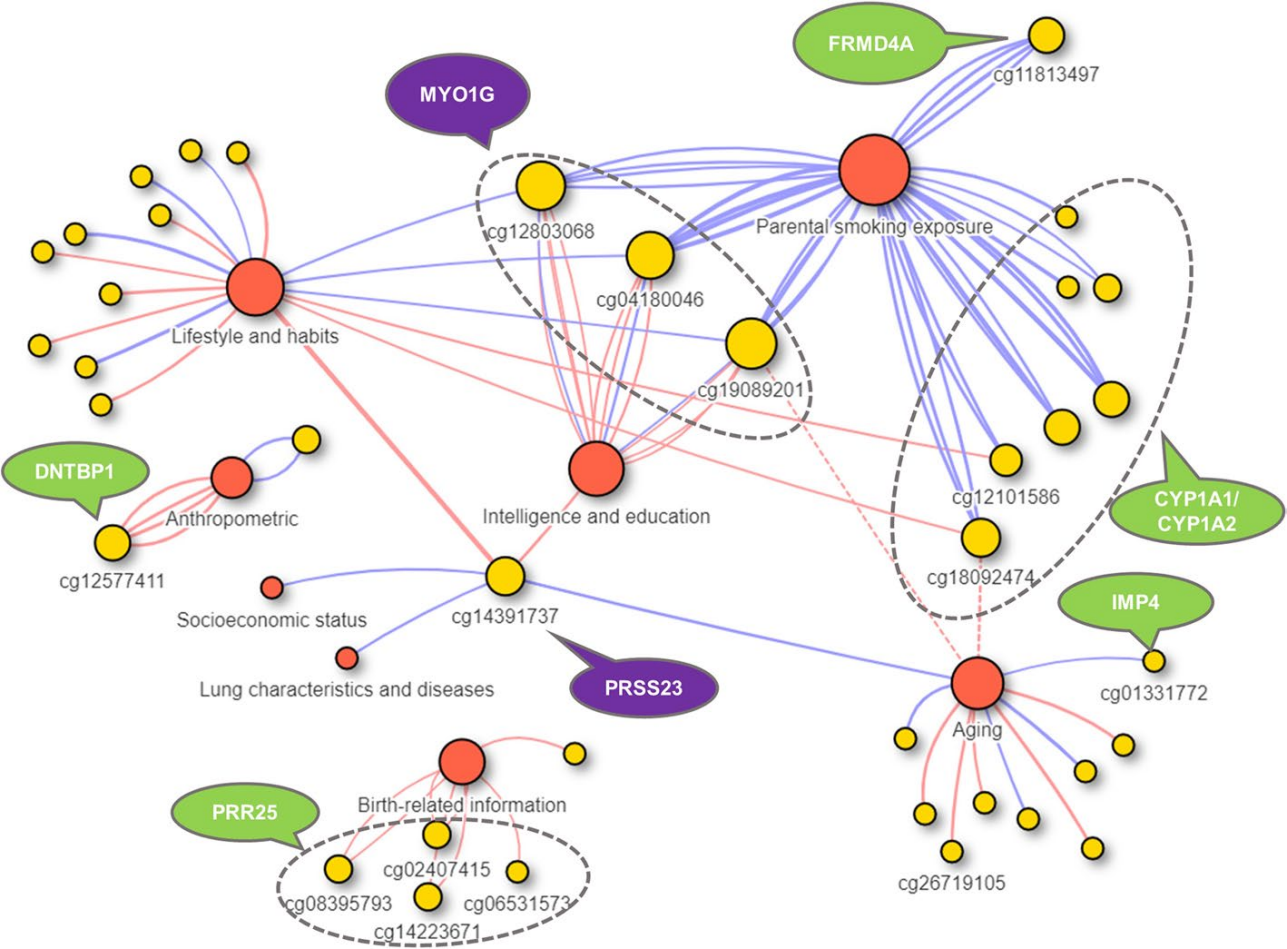
The 92 high-confidence associations between POE-CpGs and phenotypes. Red circles: phenotypic categories. Yellow circles: POE-CpGs. Dotted grey ovals: POE-CpGs located within the same gene are grouped in the same oval. Gene names with purple background: the POE-CpGs within that gene were associated with phenotypes from multiple trait categories. Lines: each line represents a significant pair, with red and blue lines representing negative and positive correlations, respectively; the two red dotted lines mark that, although the associations between cg18092474 and cg19089201 with mother’s lifespan reached statistical significance, they were likely introduced by the association between maternal smoking and mother’s lifespan

The 92 high-confidence associations included the cases where a single POE-CpG was associated with the phenotypes from multiple trait categories (Fig. 5, Additional file 2: Table S2). For example, the hypermethylation of cg14391737, a POE-CpG located in a CpG shore and an intron of the gene *PRSS23* (Serine Protease 23), was simultaneously associated with decreased smoking exposure (self), higher DNAmTL acceleration (longer age-adjusted DNAm telomere length), higher education, higher forced expiratory flow (better lung function), and higher Scottish index of multiple deprivation score (SIMD) (better socioeconomic status). For three POE-CpGs (cg04180046, cg19089201, and cg12803068) in the gene *MYO1G* (Myosin IG), the hypermethylation was associated with increased maternal smoking exposure, increased smoking exposure (self), and lower intellectual/educational level. In some cases, a single POE-CpG was associated with multiple phenotypes within a same phenotypic category (Fig. 5, Additional file 2: Table S2). For instance, multiple POE-CpGs in the gene *PRR25* were associated with both maternal and paternal ages when the offspring was born. A POE-CpG in the gene *DNTBP1* was associated with anthropometric traits (body fat composition, body mass index, weight, and waist). Seven POE-CpGs in the gene *CYP1A1/CYP1A2* and one POE-CpG in the gene *FRMD4A* were associated with maternal smoking exposures (both current and before pregnancy). Conditional analyses indicated that the multiple associations of these POE-CpGs were not driven by the socioeconomic status (measured as SIMD) (Additional file 2: Table S6).

### The atypical POE-CpGs synchronized as co-methylation modules which were associated with aging

We next hypothesized that POE-CpGs could be associated with phenotypes through co-methylation networks and that the modules constructed from the POE-CpGs belonging to different subgroups (atypical vs. typical) could display distinct phenotypic association features. To test these hypotheses, we identified the co-methylation modules for the atypical and typical POE-CpG groups, respectively. The co-methylation modules were initially constructed in the discovery and replication datasets independently, after which “consistent modules” across datasets were identified (see “Methods”). For each “consistent module,” the principal components (PCs) of constituent CpGs’ methylation levels were calculated (see “Methods”). The PCs that both had a sum of squared (SS) loadings larger than one and explained more than 5% of the methylation variation in the modules were used in module-based phenome-wide association tests.

**Fig. 6:**
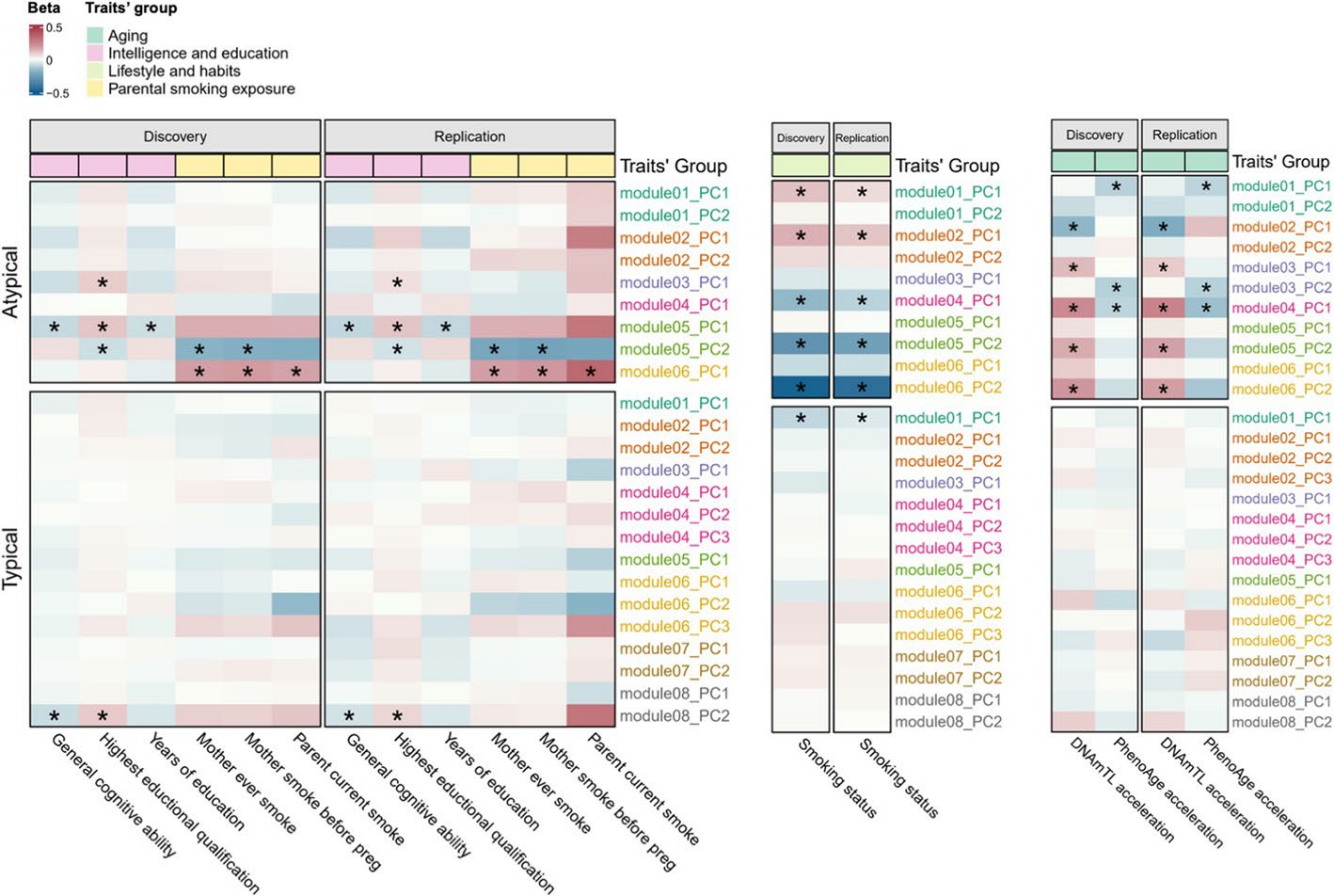
Associations between POE co-methylation module PCs and phenotypes. Only results for the phenotypes with at least one significant result are shown. The colors of the cells indicate the beta coefficient of the association. “*”: replicated associations (after Bonferroni correction). The three panels display the results for three different types of phenotypes separately, as the PCs sets used in the association tests were slightly different across phenotypes. This is because different covariates were used in pre-corrections for methylation levels when generating the module PCs (e.g., to test for the association with smoking status, the smoking variables should not be pre-corrected in methylation levels. The same applies to the aging phenotypes for which age was not pre-corrected). Left: phenotypes except for aging phenotypes and smoking status; middle: smoking status; right: aging phenotypes

The results showed that the POE co-methylation networks were highly reproducible across the discovery and replication datasets (Additional file 1: Fig. S2, Additional file 2: Table S7). Six and eight “consistent modules” were identified for the atypical and typical POE-CpG groups, respectively (Additional file 2: Table S8). Using the discovery dataset, 30 and 5 significant module-PC-phenotype associations were identified (Bonferroni method adjusted *P* < 0.05) for the atypical and typical POE-CpG modules, respectively. Using the replication dataset, 23 (77%) and 3 (60%) of the significant associations were statistically replicated for the atypical and typical POE-CpG groups, respectively (Additional file 2: Table S9, Fig. 6). For the atypical POE group, multiple co-methylation modules were associated with aging phenotypes (DNAmTL acceleration and PhenoAge acceleration) and smoking status; other associations involved intelligence/education traits and maternal smoking exposures (Fig. 6). For the typical POE group, weak associations were detected in smoking status and intelligence/education phenotypes (Fig. 6).

### An aging-associated atypical POE co-methylation network (module) whose internal methylation connectivity increases with age

The POE co-methylation modules’ association with aging implied that POE-CpGs could associate with aging in an interconnected and synchronized way. Within the aging-associated modules, would the internal methylation connectivity alter during aging? To address this, we stratified samples into six different age groups: 18 ~ 27 years (y), 28 ~ 37y, 38 ~ 46y, 47 ~ 56y, 57 ~ 65y, 66 ~ 94y. For each aging-associated POE co-methylation module, the mean and the variance of the methylation connectivity across constituent CpGs were calculated for each age group and compared across groups.

**Fig. 7:**
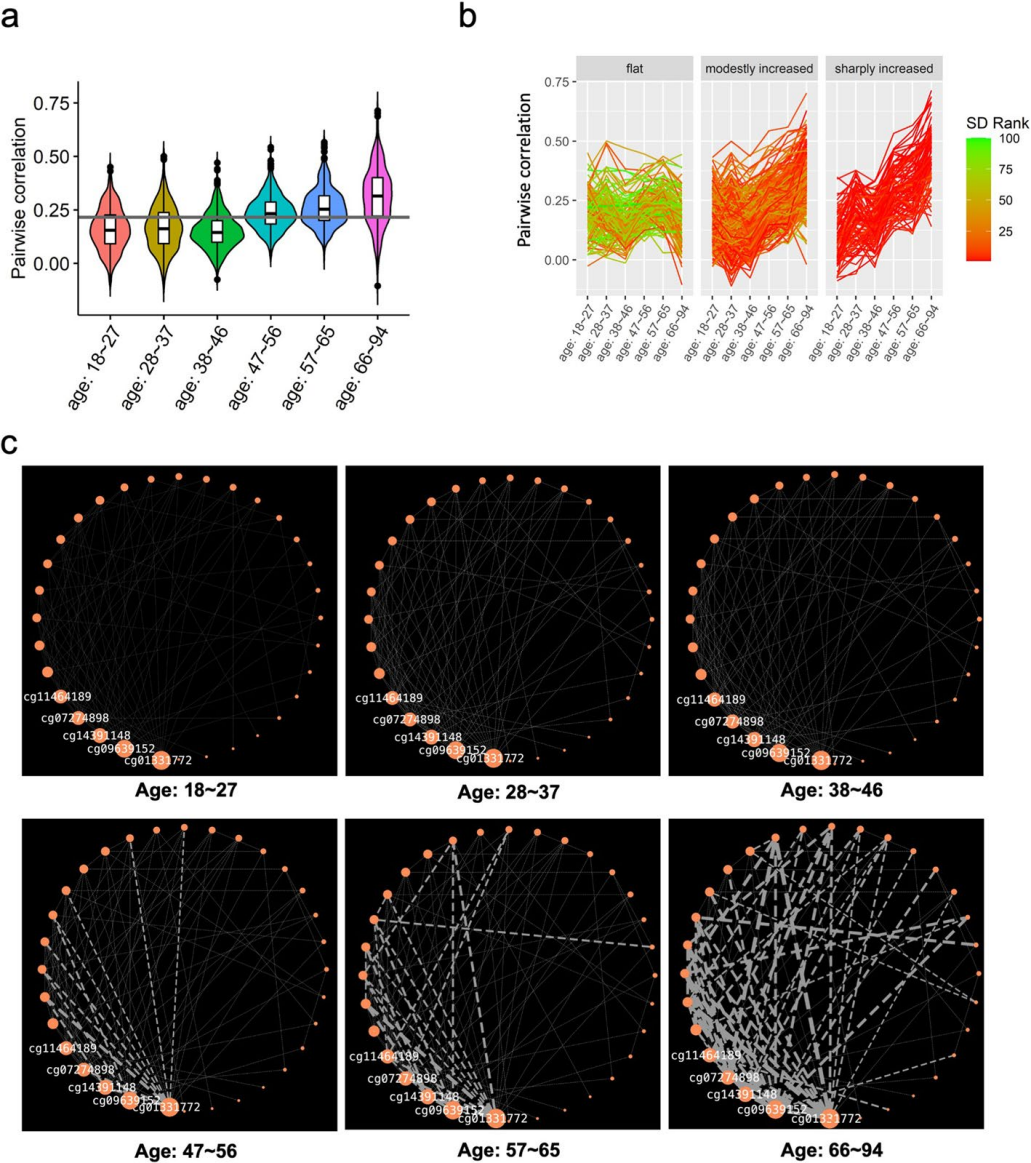
Atypical POE module 3, the module whose internal connectivity increased with age. **a** Pairwise between-CpG correlation of constituent CpGs of atypical POE module 3 across different age groups. Horizontal line: the mean of the methylation correlation across all age groups. **b** Three subclusters identified within atypical POE module 3 based on longitudinal trajectory of module connectivity; each line connects the methylation correlation value of a pair of POE-CpGs in different age groups; the color of the line corresponds to the rank of the standard deviation based on the connectivity of POE-CpG pairs across different age groups. **c** Methylation connectivity of POE-CpG pairs belonging to the “sharply increasing cluster” in each age group. Orange nodes represent POE-CpGs, the size of the orange nodes is scaled by degree centrality (the IDs of the top 5 hub CpGs are shown), the width of the edges is scaled by pairwise correlation in samples from each age group. Only edges connecting CpGs pairs with an absolute value of correlation larger than 0.4 are shown

The results revealed that the mean of the methylation connectivity within the atypical POE module 3 progressively increased with age (mean of the absolute correlation: 0.163 (0.156–0.171) for the 18–27y group and 0.315 (0.305–0.326) for the 66–94y group (Fig. 7a, Additional file 2: Table S10). The variance of the methylation connectivity of the same module also increased with age, suggesting existence of subgroups (Fig. 7a, Additional file 2: Table S11). Indeed, based on the longitudinal trajectory of the within-module methylation connectivity, our clustering analyses revealed that the co-methylated CpG pairs within this module could be further divided into three clusters: a relatively flat cluster (c1), a modestly increasing cluster (c2), and a sharply increasing cluster (c3) (Fig. 7b). In the sharply increasing cluster (c3), five CpGs (cg01331772, cg09639152, cg14391148, cg07274898, cg11464189) acted as the “hubs” that displayed the highest centrality, connected with the most other CpGs (Additional file 2: Table S12). The methylation connections radiated from the five hub POE-CpGs significantly increased the strength during aging, with the strongest connectivity detected in the oldest age group (66–94y) (Fig. 7c, Additional file 2: Table S13). In contrast, none of other constituent CpGs of this module displayed such significant alteration of connectivity strength with age (Additional file 2: Table S13). These results revealed the central role of the five hub CpGs in driving the increased methylation connectivity pattern of the atypical POE module 3 during aging, suggesting the increased importance of the module and the five hub CpGs at older age groups.

Although the five hub CpGs are in different chromosomes (Additional file 1: Fig. S3), they are all located within functional regulatory regions as reported by the Roadmap Epigenomics project [37]: cg01331772 and cg07274898 are located in promoters (active TSS); cg09639152, cg14391148, and cg11464189 are located in enhancers (mostly bivalent enhancers) (Additional file 1: Fig. S3, Additional file 2: Table S14), pointing towards the potential influence of their methylation variation on the expression of nearby genes. Intriguingly, four of the five hub CpGs (Additional file 2: Table S14) are located within or nearby a gene encoding a protein that physically interacts with the amyloid beta (A4) precursor protein (APP) in vitro (6.3 fold enrichment, *P*_fisher_ = 5.6 × 10^−4^) [38].

Among the four hub CpGs located within/near the APP-interactive genes, cg01331772 displayed the highest centrality (Fig. 7c, Additional file 2: Table S12) and the strongest elevation of methylation connectivity in the sharply increasing cluster (c3) in the comparison between the youngest and oldest age groups (Additional file 2: Table S13). This CpG is located in a promoter and is 987 bp downstream of the gene *CCDC115* (Coiled-Coil Domain Containing 115) and 4791 bp upstream of the gene *IMP4* (IMP U3 Small Nucleolar Ribonucleoprotein 4) (Additional file 1: Fig. S3). In blood, the methylation level of this CpG was positively associated with the mRNA expression of *IMP4* (*P*_eQTM_ = 9.7 × 10^−7^), as reported by a recent eQTM (expression quantitative trait methylation) study [39]. In brain, the methylation level of cg01331772 and the mRNA expression of *IMP4* were genetically positively correlated in our OMIC-based SMR analysis (Beta_SMR_ = 0.35, *P*_SMR_adjusted_ = 8.4 × 10^−4^, *P*_HEIDI_unadjusted_ = 0.1). For this CpG, the brain-blood methylation correlation was relatively high (rho = 0.54, *P* = 0.01, Additional file 1: Fig. S4) [40], suggesting that the methylation of cg01331772 in blood could be indicative for expression of *IMP4* in brain tissues. Notably, *IMP4*’s mRNA expression is significantly lower in Alzheimer’s disease (AD) patients as compared to controls in both temporal cortex (*P* = 0.003) and prefrontal cortex (*P* = 2.6 × 10^−6^), the two most relevant brain regions for AD pathogenesis (Additional file 1: Fig. S5) [41–43]. Putting these observations together, increased methylation of the hub CpG cg01331772 in blood may imply higher expression of *IMP4* in AD-susceptible brain tissues, which can be protective for AD.

**Fig. 8:**
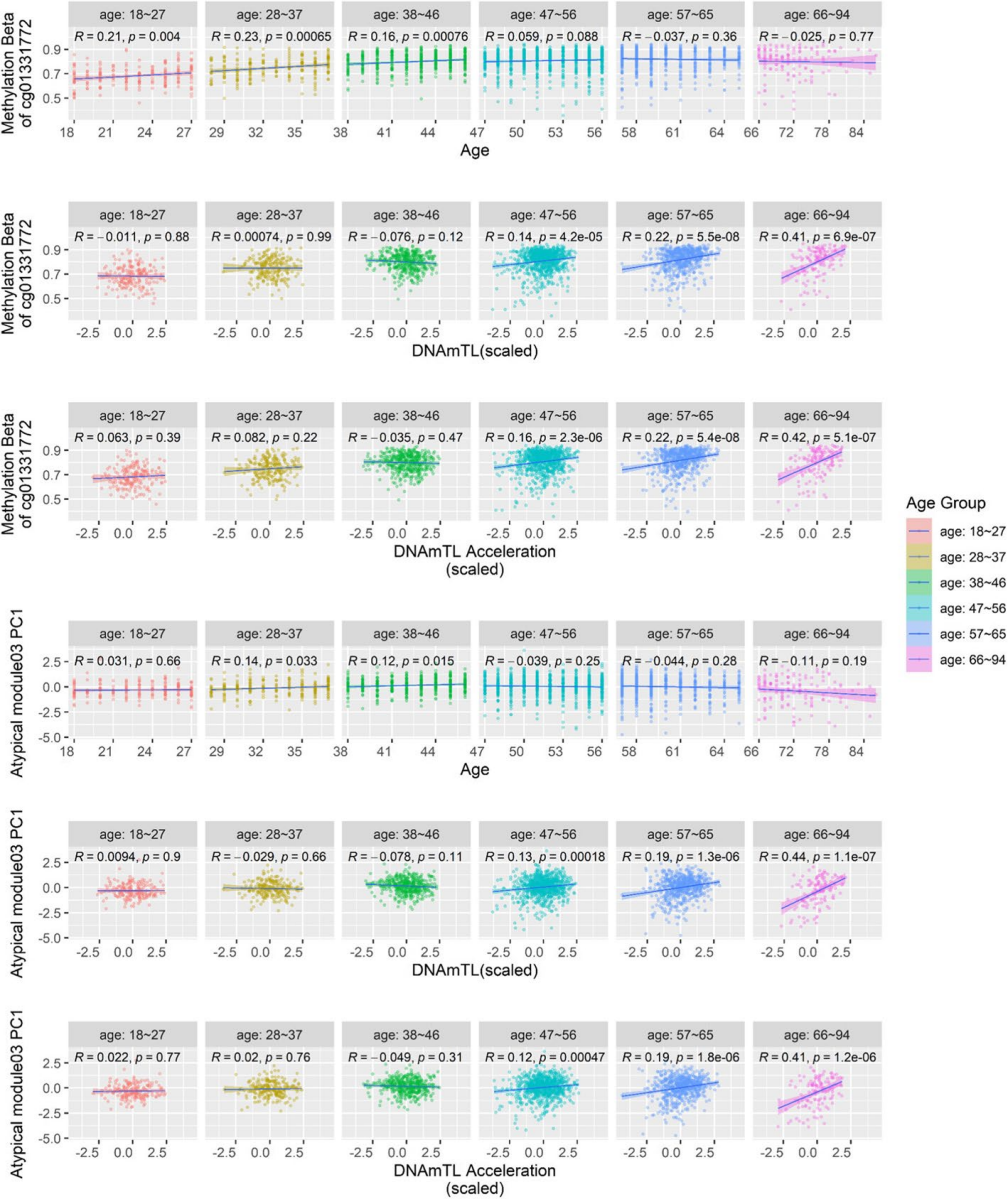
The atypical POE module 3 and its hub POE-CpG cg01331772 associations with age, DNAmTL, and DNAmTL acceleration in different age groups. Each column represents the results using samples from one age group (18–27y, 28–37y, 38–46y, 47–56y, 57–65y, 66–94y). The first three rows represent the association between cg01331772 and the three aging-related phenotypes, with the *y*-axis indicating the methylation level (beta values) of the locus; the last three rows represent the association between the top PC of the atypical POE co-methylation module 3 and the three aging-related phenotypes in different age groups

Interestingly, the associations between the hub CpG cg01331772 and aging dramatically changed cross different life stages. The PC1 of the atypical POE module 3, explaining 28.7% of the methylation variance within that module and having a positive loading from cg01331772 (Additional file 2: Table S15), displayed a similar association pattern with aging. In brief, using the samples from the full age spectrum in the GS:SFHS (18–94y), at the single-CpG level, we found that the hypermethylation of cg01331772 was associated with older chronological age (Additional file 2: Table S16) and longer age-adjusted DNAmTL (higher DNAmTL acceleration. Additional file 2: Table S2); at the modular level, the PC1 of atypical POE module 3 displayed similar association patterns (Additional file 2: Table S9). Why would the methylation of cg01331772 and the PC1 of atypical POE module 3 increase with chronological age while displaying a positive association with DNAmTL acceleration at the same time? The seemingly contradictory observations were disentangled by our age-stratified analyses. We found that starting with the youngest adult years (18–27y), the methylation of cg01331772 significantly increased with age, but the slope decreased to an insignificant level after the middle age was reached (Fig. 8). In contrast, no association between cg01331772 and DNAm-predicted telomere length was observed until middle age, after which a positive association started to arise and became much stronger in older age groups (Fig. 8). As a consequence, a significant interactive effect between chronological age and cg01331772’s methylation effect on DNAmTL acceleration was detected (*P*_interaction_ = 2.2 × 10^−8^), whereby the methylation of this CpG only manifested significant positive association with DNAmTL acceleration in old age groups (Fig. 8). Similar association patterns were observed for the PC1 of the atypical POE module 3 (Fig. 8). These combined results revealed the importance and the complexity of the role of the POE co-methylation networks and their hub POE-CpGs in human aging.

### High levels of methylation heterogeneity and increased epigenetic drift (information loss with age) of the atypical POE-CpGs

As mentioned above, both single-CpG- and network-based analyses supported the special link between POE-CpGs (the atypical group in particular) and aging. We next examined whether those CpGs manifested additional aging-related features. In the DNA methylation context, Shannon entropy measures the level of methylation heterogeneity: the higher the Shannon entropy is, the higher the heterogeneity is and the less predictable the methylation condition in a cell population is [5, 44, 45]. Shannon entropy is maximized at intermediate methylation levels (Beta = 0.5) and minimized at extreme methylation levels (Beta = 0 or 1). It has been known that aging was accompanied with an increased epigenetic drift (the loss of information stored in the epigenome), reflected as the age-related increment of average methylation Shannon entropy for the epigenome as a whole, or for a few aging-related functional CpG sets with a faster drift rate [5, 44, 45]. Here, we compared the Shannon entropy for POE-CpGs, in particular for those belonging to the atypical group, epigenetic clock CpGs and the rest of the epigenome.

**Fig. 9:**
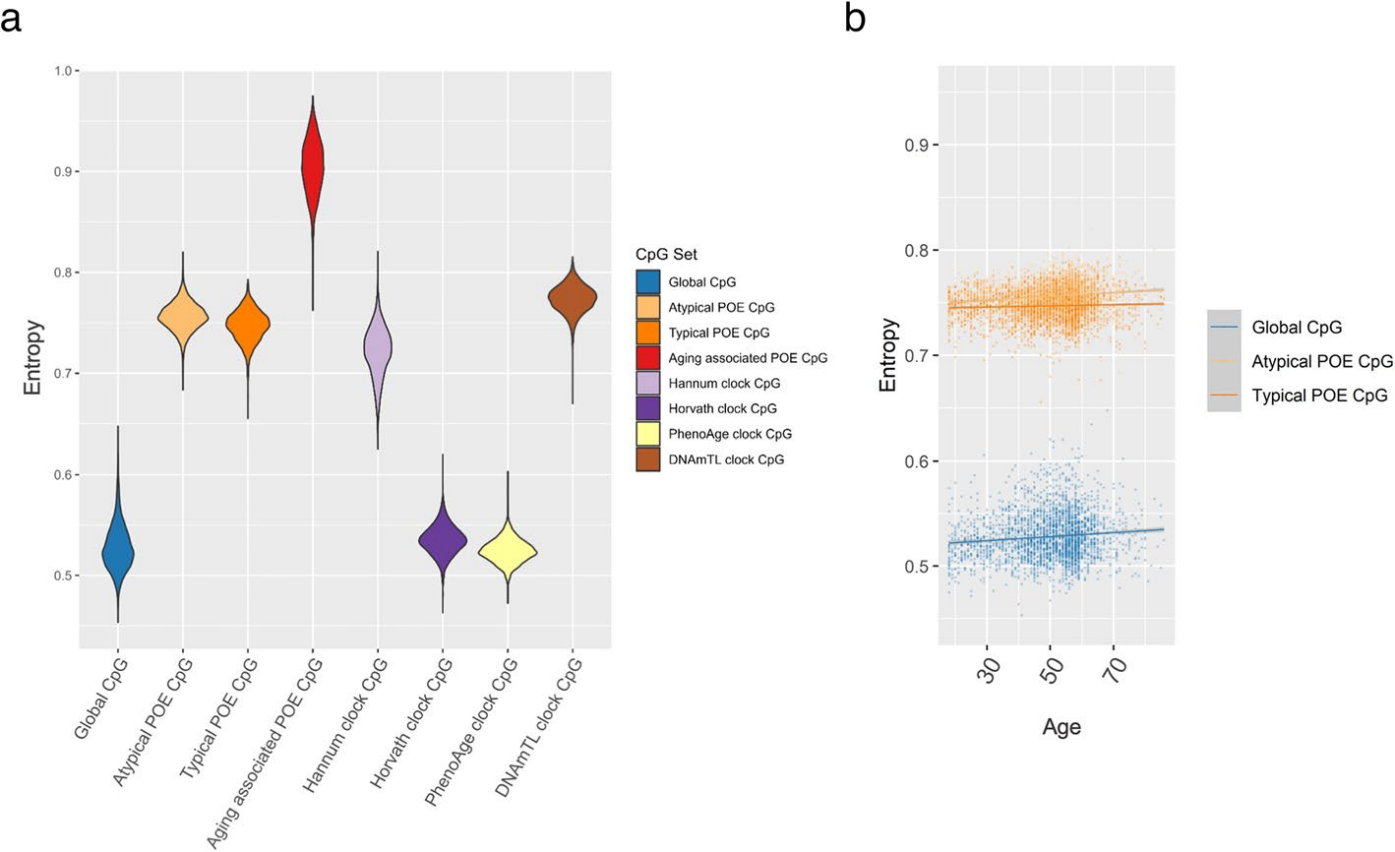
Shannon entropy of POE-CpGs and CpGs from other categories. **a** A violin plot showing the distributions of the Shannon entropy of CpGs belonging to different categories. **b** A scatter plot showing the increment of the Shannon entropy with age for POE-CpGs and the global CpGs

The results showed that taking POE-CpGs as a whole, their Shannon entropy was significantly higher than the global level of the methylome, higher than the Horvath clock and Hannum clock CpGs and slightly lower than the DNAmTL clock CpGs (Fig. 9a, Additional file 2: Table S17). After we stratified POE-CpGs into subgroups, the atypical POE group’s Shannon entropy was significantly higher than that of the typical group. The aging-associated POE-CpGs displayed higher Shannon entropy than the POE-CpGs without an association with aging (Fig. 9a, Additional file 2: Table S17). In terms of epigenetic drift (information loss) with age, Shannon entropy of all CpG groups significantly increased with age, with the atypical POE-CpG group displaying faster information loss with age as compared to the typical POE-CpG group and the global methylome (Fig. 9b, Additional file 2: Table S18).

### Intrinsic connection between the clock CpGs and the atypical POE-CpGs

**Fig. 10:**
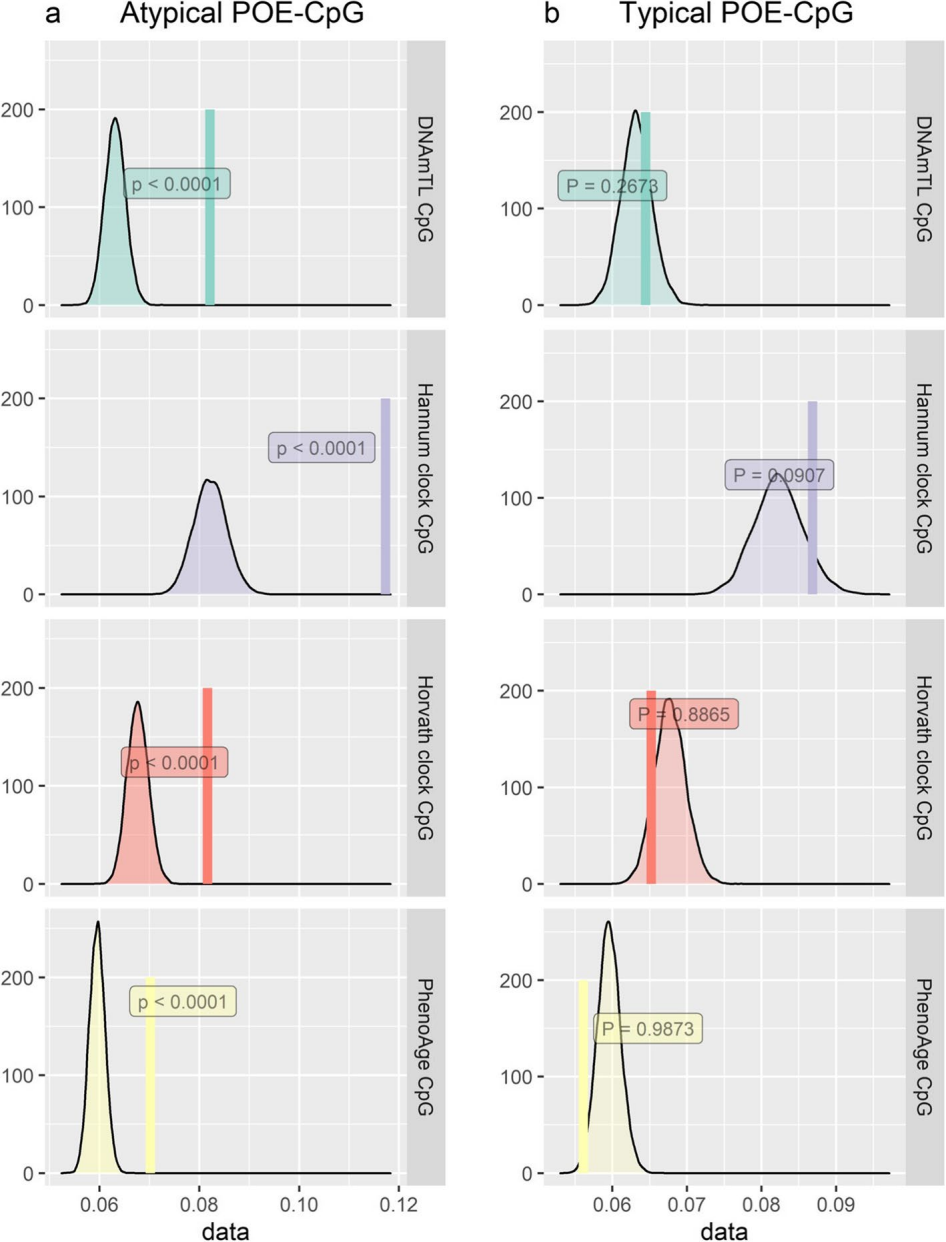
Permuted and observed correlation between POE-CpGs and constituent CpGs of epigenetic clocks. The smoothed histogram represents the null distribution of the absolute correlation values created using the permuted (*N*_permutation_ = 10,000) datasets. The vertical line represents the observed value. The *P* values represented the statistical significance obtained from the permutations

Given the shared high Shannon entropy feature both for the POE-CpGs and clock CpGs, we wondered whether the POE-CpGs and clock CpGs are intrinsically connected. To address this, a circular permutation approach was applied to test whether the atypical/typical POE-CpGs were more correlated with the clock CpGs compared with randomly selected CpG sets of the same size drawn from the methylome. The results revealed a significantly higher correlation between the atypical POE-CpGs and the constituent CpGs of all the four popular clocks when compared to the randomly drawn CpG sets, whereas this was not observed in the typical POE-CpG group (Fig. 10).

## Discussion

In this study, we systematically examined the associations of the POE-influenced methylome (POE-CpGs) with adult aging, early/late environmental exposures, and health-related phenotypes. The single-CpG-based analyses identified replicated and enriched methylation associations with lifestyle (smoking status), aging (DNAmTL acceleration), parental (maternal) smoking exposure, and intelligence phenotypes in the atypical POE-influenced regions. The co-methylation analyses indicated that at least a proportion of the atypical POE-CpGs were associated with these phenotypes in a modularized way. We additionally reported the age-related increment of internal methylation connectivity in an aging-associated atypical POE co-methylation module. For that module, we identified the hub POE-CpGs that likely drive the increment of the connectivity. We also uncovered the dynamic aging-association patterns of the module and its top hub CpG across different life stages. Finally, compared to the rest of the methylome, the atypical POE-CpGs displayed high levels of methylation heterogeneity, fast information loss with age, and high methylation correlation with clock CpGs, which further provided evidence for the special link between the atypical POE-influenced methylome and human aging.

At the single-CpG level, we found that the atypical POE-influenced methylome was sensitive to both early-life factors such as maternal smoking exposure and parental age when the offspring was born, and later-life exposures such as smoking and alcohol consumption. Meanwhile, the atypical POE-CpGs were also strongly associated with aging and health-related phenotypes such as intelligence in adulthood (Figs. 3 and 4). Importantly, we detected some cases where the same single POE-CpG was simultaneously associated with both environmental exposure (such as maternal smoking exposure or lifestyle), adult aging, and/or with health-related phenotypes (such as intelligence). Our observation of the associations between cg14391737, an intronic POE-CpG located of the gene *PRSS23*, with smoking status and forced expiratory flow (Fig. 5), was in line with the previous MWAS papers that identified cg14391737 as a smoking- and lung cancer-associated CpG [46, 47]. Here, we uncovered its additional associations with education, socioeconomic status, and DNAmTL acceleration (all of these associations are identified after smoking effects were accounted for). Our observation that multiple CpGs within the gene *MYO1G* were associated with maternal smoking exposure, smoking status, and the highest educational qualification, was consistent with previous studies [48–50]. Importantly, we uncovered the new associations of those early and late environmental-sensitive CpGs in *MYO1G* with multiple intelligence measurements in adults (Additional file 2: Table S2). These results supported well our hypothesis that the POE-influenced epigenome could act as a hub in the interplay of early/late-life exposures, adult health, and adult aging.

At the network level, we found that the methylation levels of a proportion of POE-CpGs fluctuated jointly as co-methylation modules (both in *cis* (close proximity on the chromosome) and *trans* (distant proximity on the chromosome)). Consistent with the results from the single-CpG-based analyses, the module-level results revealed the association of the shared methylation variation of multiple atypical POE-CpGs with aging, smoking, maternal smoking exposure, and intelligence. These results suggested that the aging-associated atypical POE-CpGs can function in a modularized way and that early and late environment may influence the atypical POE-CpGs in groups rather than individually.

The aging-associated POE co-methylation networks were not stable throughout the life. We found that the atypical POE module 3, one of the aging-associated POE co-methylation modules, displayed increased connectivity when humans get older (Fig. 7). Five hub POE-CpGs were identified for their central role in driving this change, and intriguingly, the majority of them appeared to link to APP-interacting proteins. In particular, the overall module centrality and the aging-associated connectivity change were most prominent in cg01331772, a promoter CpG that was likely capable of regulating the expression of *IMP4*, the gene both interacting with APP and displaying significant downregulation in AD patients in two AD-relevant brain regions (Additional file 1: Fig. S5). These findings coincided with a previous finding suggesting that at methylome-wide level, the aging-associated co-methylation module was enriched for promoter CpGs located nearby genes downregulated in early disease stage of AD [51]. Our results suggested the central role of *IMP4*’s regulatory CpG cg01331772 in the POE-related modularized methylation alteration during the aging process.

The complexity of the role of the atypical POE module 3 and its hub CpG cg01331772 in human aging can be further revealed by integrating existing evidence from previous studies with our single-CpG- and network-based results. Previous studies have recognized that the methylation of cg01331772 persistently increased at early-life stage (age ≤ 10y) [52–54]. Our stratification analyses covered a wide age spectrum of human adults (18–94y) and showed that the age-associated elevation of methylation in this CpG continued until middle age. For older age groups, this CpG was no longer associated with chronological age, but surprisingly, shifted to be associated with increased DNAm-predicted telomere length (DNAmTL) and age-adjusted DNAmTL (DNAmTL acceleration), with the strongest association appearing in the oldest age group (66–94y) (Fig. 8). The PC1 of the atypical POE module 3 where cg01331772 has a positive loading also followed this pattern (Fig. 8). Previously, IMP4 has been reported as a component of telomerase whose function was to maintain/elongate telomeres [55, 56]. Here, we found that the hypermethylation of cg01331772, a likely regulatory POE-CpG for *IMP4*, was associated with longer telomeres (predicted) in older adult groups. Importantly, since cg01331772 acted as a hub CpG for an aging-associated co-methylation module that becomes highly self-connected at old age, this effect has the potential to propagate through the co-methylation network. These observations unveiled new targets (cg01331772 and other constituent CpGs of the atypical POE module 3) for future biomarker and intervention studies of aging. They also highlighted that, in order to comprehensively evaluate the multiplex role of functional CpGs such as POE-CpGs in the human aging, it is necessary to consider the effects both when CpGs act as individual sites and act as constituent members in a network. The aging-association patterns appear to be dynamic in different age groups for at least some functional CpGs. The mean methylation levels and the connectivity strength could reveal different aspects of the methylation network.

POE-CpGs also manifested other aging-related features such as a high degree of methylation heterogeneity, a fast epigenetic drift with age and a strong methylation correlation with the constituent CpGs of the four epigenetic clocks in the case of the atypical POE group. As the clock CpGs were well known for the associations with aging, here, they were used to compare with the POE-CpGs to benchmark the aging-related features of those CpGs. Previous studies have reported the genome-wide trend of loss of methylation information content (manifested as increased entropy) with age [5, 45]. A high entropy in epigenetic clock CpGs compared to the rest of the genome [57] and a positive association between methylation entropy and age acceleration [5] have also been reported. Here, our study showed that as a specialized group of CpGs, POE-CpGs (the atypical group) not only lost methylation information with age at a rate that was faster than the rest of the methylome, but also displayed unusually high methylation heterogeneity (entropy), even higher than the constituent CpGs of three popular epigenetic clocks (Horvath, Hannum, PhenoAge). The POE-CpGs’ entropy was slightly lower than that of DNAmTL CpGs when considering POE-CpGs as a whole. However, it was higher when only considering the aging-associated POE-CpGs. The high entropy feature shared between the POE-CpGs and clock CpGs inspired us to hypothesize that POE-CpGs and clock CpGs were intrinsically interconnected, given their shared association with aging. Indeed, although there were only 10 CpGs labeled as both POE-CpGs and clock CpGs, we found a much higher correlation between the atypical POE-CpGs and the clock CpGs for all of the four clocks tested, compared to the correlation with the rest of the methylome (Fig. 10). This was not observed in the typical POE-CpG group, consistent with our observation that the atypical POE-CpG group displayed much stronger and enriched associations with aging phenotypes compared to the typical POE-CpGs (Figs. 3 and 4). It is noteworthy that the clock CpGs have been known for their ability to predict aging, whereas the POE-CpGs were identified for the special heritable pattern introduced by early-life events (imprinting or early environmental influence); the shared features between the two classes of CpGs further supported the association between the atypical POE-CpGs and aging.

This study revealed substantial differences between the POE-CpGs belonging to the atypical and typical groups. Imprinting-related POE (the typical type) have been previously associated with metabolic, behavioral, and neurological traits [58]. Although our results also supported those associations (Fig. 3), we found that the methylation associations with those traits are not enriched in the typical POE regions (Fig. 4), indicating the complex mechanisms of those traits and the relatively small overall influence of imprinting for those traits at the population level. Compared to the relatively well studied imprinting-related POE methylome (the typical type), the atypical POE-influenced methylome has been largely understudied. Our study provided multi-level evidence for the environment-sensitive and aging-related features of the atypical POE-influenced methylome. The early effects from maternal smoking and maternal age when the child was born on DNA methylation were found to be highly enriched in those regions (Fig. 4). The strong associations with adult aging and other health phenotypes further revealed the dynamic features of the atypical POE-CpGs throughout the life. These results emphasized the need for future research on this sensitive and flexible part of the methylome.

There are limitations in this study. First, the associations we reported were discovered and replicated in a Scottish population. The discovery dataset in this study used overlapping samples with the study that reported the imbalanced methylation features of POE-CpGs [20]. Future studies are needed to replicate our findings in other populations. Second, survival bias could influence the estimates of the methylation connectivity for the atypical POE module 3 in old age groups, given the cross-sectional feature of our samples. Although the methylation connectivity of that module has already started to increase at young age (Fig. 7), which suggests that the overall increasing trend is less likely to suffer from the survival bias issue, future longitudinal data will help to validate our findings in older age groups. Third, the aging-associated methylation dynamics can be confounded by varied cell proportions of rare cell types. Although we accounted for cell count effects by pre-adjusting or jointly fitting estimated proportion of major blood cell types as covariates, the proportions of rare cell types can vary substantially across age groups but are difficult to estimate, this could confound the methylation analyses using the data generated from bulk tissues like ours. Fourth, our analyses on effects from early environmental exposures on POE-CpGs were largely limited to parental smoking. Future analyses using the samples with richer and higher resolution records of early environmental exposures would allow a more comprehensive evaluation of effects from early environmental exposures and lifetime consequences. Fifth, POE-CpGs’ associations in the offspring with heritable traits such as intelligence could tag the genetic-mediated environmental effects from parental behaviors, which have a genetic component relating to the parents’ intelligence. Future POE studies accounting for the genetic nurture will be of great interest to disentangle these effects. Finally, although longer telomeres (and the higher telomere length acceleration) in non-tumor tissues are usually considered protective, there is also evidence suggesting that longer telomeres can be associated with higher risk of cancer [59]. We suggest that the conclusions regarding longevity from our DNAmTL acceleration analyses should be made with caution and that future studies to investigate the association between POE-CpGs and longevity directly are warranted.

## Conclusions

Our phenome-wide human methylation analyses identified strong and enriched associations between the atypical POE-influenced methylome and adult aging, and between the atypical POE-influenced methylome and early/late exposures at both single-CpG and network levels. The shared high methylation heterogeneity features and the intrinsic connections between the atypical POE-CpGs and the clock CpGs were also revealed. The identified single POE-CpGs and POE co-methylation modules provided new targets for future biomarker and intervention studies and added novel supporting evidence for the “early development of origin” hypothesis for adult aging.

## Methods

### Population samples

Generation Scotland: Scottish Family Health Study (GS:SFHS) is a family-based population cohort with extensive health-related phenotypes, records of environmental exposures, and genome-wide genotypes collected for 19,994 Scottish participants [34, 60]. Genome-wide DNA methylation data (whole blood) was also available for 9526 participants [35]. The methylation data was produced and processed independently in two batches, for 5081 participants in 2016–2017 (batch 1) and 4445 participants in 2019 (batch 2). All participants in batch 2 were genetically unrelated (relatedness < 0.05) to each other and to the participants in batch 1. We used batch 1 as the discovery dataset and batch 2 as the replication dataset in downstream analyses.

### DNA methylation data

The discovery and the replication datasets were generated, processed, and quality controlled in a similar way [61] based on a pipeline proposed previously [20, 62]. In brief, the methylation signals for 866,836 sites were measured using the Illumina Infinium MethylationEPIC array (http://support.illumina.com) for the whole blood sample of each participant. The “estimateCellCounts” function in the R package minfi was used to estimate the proportion of major blood cell types: B-lymphocytes, natural killer cells, monocytes, granulocytes, CD4 + T-lymphocytes, and CD8 + T-lymphocytes [63]. The R packages shinyMethyl and meffil were used for quality control [64, 65]. The performance of control probes, signal intensity, and the consistency between the registered and predicted sex were used to identify outlier samples and probes. In addition, samples were removed if more than 0.5% of measured sites had a detection *p* value > 0.01. Probes were removed if more than 1% of samples were missing or had a bead count ≤ 3, or if they had cross-hybridization or overlapped with any common SNP (MAF ≥ 0.01) in the European population [66]. After the quality control, normalization was performed using the “ssNoob” method in the R package minfi [67]. As described before [20], the normalized M values were adjusted, using a linear mixed model, for technical variables including sentrix variables (id and position), processing batches, clinics, appointment variables for the blood extraction (date, weekday, and year), and the top 20 PCs calculated from the control probes [62]. Resultant residuals were available for 734,436 methylation sites which were used in downstream analyses.

### Phenotype data

The phenotypes in GS:SFHS consisted of 142 variables in 15 categories (Additional file 2: Table S19). Among them, birth and maternity variables were obtained through data linkage with historic Scottish birth cohorts for a subset of GS:SFHS participants [68]. The aging category comprised four variables, including mother’s/father’s lifespan and two epigenetic-based measurements for biological aging (PhenoAge acceleration and DNAmTL acceleration) [6, 7]. The two acceleration measurements were calculated as the residuals from the regressions of PhenoAge, an epigenetic clock designed to predict healthspan (phenotypic age) [6], and DNAmTL, an epigenetic clock designed to predict telomere length [7], on age and age^2^. A positive PhenoAge acceleration corresponds to excessive biological aging among individuals of the same chronological age, whereas a positive DNAmTL acceleration corresponds to the additional (longer) telomere length after accounting for chronological age. The phenotypic correlation between the four aging measures is shown in Additional file 1: Fig. S6. The quantitative traits with a skewed distribution were log transformed with base 10. The measurements that fall outside of four standard deviations from the mean were identified as outliers and thus removed. More details of the phenotypes are provided in Additional file 2: Table S19.

### Phenome-wide association analyses for the POE-influenced methylation sites

The phenome-wide association analyses for individual POE-CpG sites were performed using the MOA model:

$$\mathrm{MOA}\;\mathrm{model}\;:\;y_p=w_mb_m+\mathrm{ORM}\;\left(\mathrm{random}\;\mathrm{effect}\right)$$

As proposed by the Omic-data-based Complex trait Analysis software (OSCA) [36], the MOA model fitted an Omic-Relationship-Matrix (ORM) as a random effect jointly with the target CpG variable as a fixed effect in linear mixed models [36]. The ORM represented the epigenetic relationships between samples and was created by the “–make-orm” function in OSCA using genome-wide probes (*N* = 734,436). M values were pre-adjusted for cell proportion, appointment variables, age, age^2^, sex, and smoking variables (smoking status and pack years). Age and age^2^ were not pre-adjusted if PhenoAge acceleration or DNAmTL acceleration was the target phenotype; smoking variables were not pre-adjusted if smoking status was the target phenotype. *y*_*p*_ is the target phenotype pre-adjusted for the two random effects represented by the genomic relationship matrix (G) and the kinship relationship matrix (K) (accounting for the genetic structure in GS:SFHS), and clinic effect (as fixed effect), using the genome-based restricted maximum likelihood (GREML) method in GCTA [69]. *w*_*m*_ is the methylation level of the target CpG site. *b*_*m*_ is the target effect to be estimated.

The MOA approach was applied to each of the POE-CpG and phenotype pairs. Since the pre-adjustment did not converge for 9 out of the 943 POE-CpGs, we only included the remaining 934 POE-CpGs in this analysis. The false discovery rate (FDR) method was used to correct for multiple testing in both the discovery (*N*_tests_discovery_ = 934 × 142 = 132,628) and replication stages (*N*_tests_in_replication_ = *N*_significant_pairs_in_discovery_ = 115).

For the replicated results, the *CpG outcome model* was used to validate the robustness of the MOA results:

$$\mathrm{The}\;\mathrm{CpGoutcome}\;\mathrm{model}\;:y_m=w_{\mathrm{covariates}}b_{\mathrm{covariates}+w_pb_p}$$

In contrast to the MOA models, the CpG outcome model is a linear fixed effect regression model that takes methylation levels of the target CpG sites as the dependent variable and the target phenotype values as the independent variable, with methylation-related biological covariates being jointly fitted in the model to avoid having to pre-adjust for those covariates. *y*_*m*_ is the methylation level of the target CpG sites after pre-adjusting for the G and K components as random effects (to account for genetic structure) and clinic effect as a fixed effect using GREML [69]. *w*_covariates_ is a matrix for covariates including blood cell proportions, appointment variables, age, age^2^, sex, and smoking variables (age and age^2^ were not fitted when PhenoAge acceleration or DNAmTL acceleration was the target phenotype; smoking variables were not fitted when smoking status was the target phenotype). *b*_covariates_ is the effects from covariates. *w*_*p*_ is the target phenotype and *b*_*p*_ is the target effect to be estimated. The FDR method was used for multiple testing correction (*N*_test_discovery_ = 98, *N*_test_replication_ = 97). We only considered the results that were statistically significant and replicated in both the MOA model and the CpG outcome model as high-confidence results.

### Comparison of the phenotypic associations with the POE vs the non-POE methylome

This analysis was to test whether for a given phenotype its association with POE-CpGs was significantly stronger than its associations with the rest of methylome. In brief, methylome-wide association studies (MWASs, *N*_CpG=_734,438) were performed using the same MOA approach for the phenotypes associated with at least one POE-CpG. The Wilcoxon rank sum test was then applied to each phenotype to test whether the *P* values of the POE-CpG-specific methylation-phenotype associations ranked significantly differently from the *P* values of associations for the rest of methylome. The Bonferroni method was applied to adjust for multiple testing correction (*N*_test_ = 48).

### Identification of modules of co-methylated CpGs in the POE-influenced methylome

Weighted gene correlation network analysis (WGCNA) was applied to identify the modules of co-methylated POE-CpGs [70]. Before constructing the modules, the methylation levels of POE-CpGs were pre-corrected by cell proportions, appointment variables, age, age^2^, sex, and smoking variables.

Given the differentiated features of the typical and atypical POE-CpGs, co-methylation modules were constructed for the typical type (*N* = 560) and the atypical type (*N* = 383) of POE-CpGs separately, and for the discovery (only unrelated samples (relatedness < 0.05) were used in network construction, *N* = 2583) and the replication datasets separately. The “soft thresholding power” parameter was optimized to allow identification of both tightly connected CpG clusters such as those in *cis* (for example, CpGs from the same island) and modestly connected CpG clusters such as those in *trans* (for example CpGs in different chromosomes). In more detail, a recursive process was applied as follows: (1) all typical/atypical POE-CpGs were used to fit the “PickSoftThreshold” function and construct networks. In this step, the picked threshold was high and only tightly connected CpGs were assigned to modules. (2) For each module identified by step 1, only one index CpG that displayed the highest correlation with other CpGs was retained in every 10-kb window. (3) Steps 1 and 2 were repeated until no more typical/atypical POE-CpGs were removed. (4)The retained set of typical/atypical POE-CpGs was used to re-fit the PickSoftThreshold function. At this stage, the optimized soft thresholding power could be estimated. (5) We used this optimized parameter (equal to three) to construct full networks using all typical/atypical POE-CpGs. The smallest number of CpGs for a module was set to 8. Other parameters were set to the default ones.

### Matching POE co-methylation modules across the discovery and replication datasets

Since the POE co-methylation modules were identified independently in the discovery and replication datasets, we matched the modules in the two datasets using following steps: (1) for any two modules, one from the discovery dataset and one from the replication dataset, the overlap rate was calculated as the number of CpGs in the intersection divided by the number of CpGs in the union. (2) All discovery-replication module pairs were ranked by overlap rate in descending order. Starting from the top pair, if the overlap rate was higher than 60%, the specific modules across datasets were successfully matched. (3) For modules identified in the replication dataset but not matched with any module in the discovery dataset in the previous step, we calculated the secondary overlap rate with each discovery module, defined as the number of CpGs in the intersection divided by the number of CpGs in the replication module. A replication module was matched to a discovery module (that is, to allow more than one replication modules to be matched to one discovery module) if the secondary overlap rate was higher than 90%. (4) The matched modules were labeled as “consistent modules,” with the shared CpGs labeled as constituent CpGs and used in downstream analyses.

### Phenome-wide association analyses for the POE co-methylation modules

#### Identification of the principal components for the POE co-methylation modules

To characterize the POE co-methylation modules, we performed principal component analyses (PCA) for the methylation levels of the constituent CpGs for each “consistent module” using the unrelated samples (relatedness < 0.05) from the discovery dataset (*N* = 2583). The estimated formula was then projected to the entire cohort to calculate the module PCs for all discovery and replication samples. This was done using the R package “psych” (https://CRAN.R-project.org/package=psych). In downstream analyses, we only used the module PCs that had a SS loading > 1 and explained > 5% of the methylation variation of the corresponding module. Similar to the single-CpG-based analyses, analyses for aging phenotypes such as the two age acceleration phenotypes and smoking status required a modified list of covariates. We therefore prepared three sets of PCs by using methylation levels pre-corrected for different sets of covariates:

- PC set 1: pre-corrected for cell proportions, appointment variables, smoking variables, age, age^2^, sex.
- PC set 2: same as PC set 1 but without pre-correcting for smoking variables.
- PC set 3: same as PC set 1 but without pre-correcting for age and age^2^.

#### Phenome-wide association tests for the POE co-methylation module PCs

A linear regression model was used to regress the module PCs on the phenotype:

$${y}_{p} = {w}_{module\_i\_pc\_j}{b}_{module\_i\_pc\_j}$$

Similar to the single-CpG-based tests, *y*_*p*_ represents the target phenotype pre-adjusted for the G and K components as random effects and clinic as fixed effect. *w*_module_i_pc_j_ is the top *i*th PC in module *j*. *b*_module_i_pc_j_ is the tested effect from the *i*th PC of module *j*. Since the methylation-related covariates have been pre-adjusted when generating the PCs (described above), we did not re-adjust for covariates at this step. The module PC set 1 (described above) was used for most association tests, except for the tests targeting smoking status (the module PC set 2 was used), and the tests targeting age acceleration phenotypes (the module PC set 3 was used). The Bonferroni method was used in the multiple testing correction (*N*_test_discovery_ = 3199, *N*_test_replication_ = 35).

### Analyses of the dynamics of the internal methylation connectivity for the aging-associated POE co-methylation modules across age-stratified groups

We stratified samples into six subgroups according to their chronological age (Additional file 2: Table S20). For each aging-associated POE co-methylation module, the connectivity among constituent CpGs was measured using the pairwise Pearson correlation of the methylation levels pre-adjusted for cell proportion, sex, appointment variables, and smoking variables. The connectivity difference between any two age groups was tested by the Wilcoxon rank sum test (paired test) using the R function wilcox.test, and the difference of the variance of the absolute connectivity across age groups was tested by Levene’s test using function “levenetest” in the R package “car”. Based on the age-dependent connectivity trajectories, subclusters within the module of interest were identified using hierarchical clustering. Cytoscape was used to calculate the node centrality and visualize the results [71].

### OMIC- and summary-data-based Mendelian randomization (SMR) analysis

SMR was applied to identify the pleiotropic associations between the methylation levels of target CpGs and the mRNA expression levels of nearby genes [72]. Brain *cis*-mQTL summary statistics were from Qi et al. [73], brain *cis*-eQTL summary statistics were from Qi et al. (2022) (unpublished, the data (BrainMeta v2) were accessed through the software SMR [72]). In this analysis, methylation was treated as the exposure and mRNA expression was treated as the outcome. The Bonferroni method was applied to correct for the multiple testing in SMR analyses. The HEIDI test was applied to distinguish pleiotropy from linkage, with a *P*_HELDI_ > 0.05 (unadjusted) indicating that the association was not due to linkage [72].

### Permutation tests for the connectivity between clock CpGs and POE-CpGs

The lists of CpGs used in the construction of two first-generation epigenetic clocks, the Hannum and Horvath clocks, and two second-generation epigenetic clocks, PhenoAge and DNAmTL, were downloaded from the original publications, respectively [4–7]. A circular permutation over the methylome was used to generate 10,000 random CpG sets of the same size as the typical/atypical POE-CpGs groups, keeping the overall correlation structure of the true POE-CpG set in the generated random sets [74]. For each clock, the average connectivity between the clock CpGs and the POE-CpGs (the true set and the permuted sets) was calculated as the mean of absolute values of the pairwise methylation correlation (Pearson method). Permutation *P* values were calculated by ranking the average connectivity of permuted sets in descending order and determining the position of the true average connectivity in the ranked list.

### Calculation of methylation Shannon entropy

In the context of DNA methylation, the Shannon entropy measures the level of methylation uncertainty (methylation heterogeneity) [5, 45, 57]. The following formula was used to calculate the Shannon entropy for a given CpG in a given sample [45]:

$$\mathrm{Entorpy}\left({\mathrm{CpG}}_{\mathrm{ij}}\right)=-{\mathrm m}_{\mathrm{ij}}*{\log_2\mathrm m}_{\mathrm{ij}}-\left(1-{\mathrm m}_{\mathrm{ij}}\right)*\log_2\left(1-{\mathrm m}_{\mathrm{ij}}\right)$$

where *m*_*i*_ is the beta value of a given CpG *i* for a given sample *j*.

### Annotation and visualization

Functional annotations for CpGs and genes were performed using ANNOVAR [75]. The R packages ggplot2 [76], ggpubr [77], ComplexHeatmap [78], and visNetwork [79] were used in the visualization of the presented results.

## Supplementary Information

**Additional file 1: Fig. S1.** Functional enrichment of associated POE-CpGs for each phenotype and each phenotypic category. **Fig. S2.** Identified WGCNA POE co-methylation modules in discovery and replication datasets. **Fig. S3.** Annotations for the genomic context of the five hub CpGs of the atypical POE module 3. **Fig. S4.** The correlation of methylation levels of cg01331772 between blood and brain. **Fig. S5.** Comparisons of IMP4's mRNA expression in different brain tissues in control and Alzheimer's disease patients groups. **Fig. S6.** Phenotypic correlations between the four aging phenotypes.

**Additional file 2: Table S1.** Discovery and replication results by MOA and CpG outcome models for the 115 associations identified in discovery dataset. **Table S2.** Discovery and replication results by MOA models for the 92 high-confidence associations between POE-CpGs and phenotypes. **Table S3.** Conditional analyses results for POE-CpGs’ association with parental smoking exposure. **Table S4.** Enrichment test results for POE-CpGs associated with specific phenotype in functional regions. **Table S5.** Per phenotype resultsfor comparisons between methylation association signals from POE regions versus non-POE regions. **Table S6.** Comparisons between results from sensitivity models using SIMD as covariates and results from the raw models. **Table S7.** Information for matched modules between discovery and replication datasets. **Table S8.** Information of constituting CpGs of POE co-methylation modules. **Table S9.** Significant and replicated associations between POE co-methylation module PCs and phenotypes. **Table S10.** Comparisons of the distribution of module connectivityof atypical POE module 3 across different age groups by Wilcox test. **Table S11.** Comparisons of variation of module connectivityof atypical POE module 3 across different age groups by Levene test. **Table S12.** The degree and closeness centrality of constituting GpGs of atypical POE module 3 in sharp increasing cluster. **Table S13.** Comparisons of connectivity radiated from each constituting CpG in aypical POE module 3 in the sharply increasing cluster between youngestand oldestgroups. **Table S14.** Annotations for the five hub CpGs of the atypical POE module 3. **Table S15.** The loading of each constituting POE-CpG in the first PC of atypical POE module 3. **Table S16.** Significant associations between POE-CpGs and chronological age. **Table S17.** Comparisons of Shannon entropy in CpGs belonging to different groups. **Table S18.** The correlations between methylation Shannon entropy and age. **Table S19.** Phenotypic information. **Table S20.** Number of samplesin each age group.

**Additional file 3.** Review history.

## Data Availability

Summary statistics supporting the conclusions of this article are included within the article and its additional files (Additional file 2: Table S1-S20). The full summary statistics for the association analyses at the single CpG and modular level are available at the following repository link: https://zenodo.org/record/7807379 [80]. The data dictionary for GS:SFHS is available at the URL: https://datashare.ed.ac.uk/handle/10283/2988 [81]. According to the terms of consent, access to DNA methylation and phenotype data in GS:SFHS needs to be approved by the GS Access Committee (https://www.ed.ac.uk/generation-scotland/for-researchers/access, mailto: access@generationscotland.org). The managed access process ensures that approval is granted only to research which comes under the terms of participant consent which does not allow making participant information publicly available.
