# Supplementary material for: Phenome-wide analyses identify an association between the parent-of-origin effects dependent methylome and the rate of aging in humans
Source: Genome Biol. 2023 May 15;24:117. doi: 10.1186/s13059-023-02953-6 (PMC10184337; doi:10.1186/s13059-023-02953-6)
Supplement: Supplementary file 1 — Additional file 1: Fig. S1. Functional enrichment of associated POE-CpGs for each phenotype and each phenotypic category. Fig. S2. Identified WGCNA POE co-methylation modules in discovery and replication datasets. Fig. S3. Annotations for the genomic context of the five hub CpGs of the atypical POE module 3. Fig. S4. The correlation of methylation levels of cg01331772 between blood and brain. Fig. S5. Comparisons of IMP4's mRNA expression in different brain tissues in control and Alzheimer's disease patients groups. Fig. S6. Phenotypic correlations between the four aging phenotypes. [file 13059_2023_2953_MOESM1_ESM.docx]

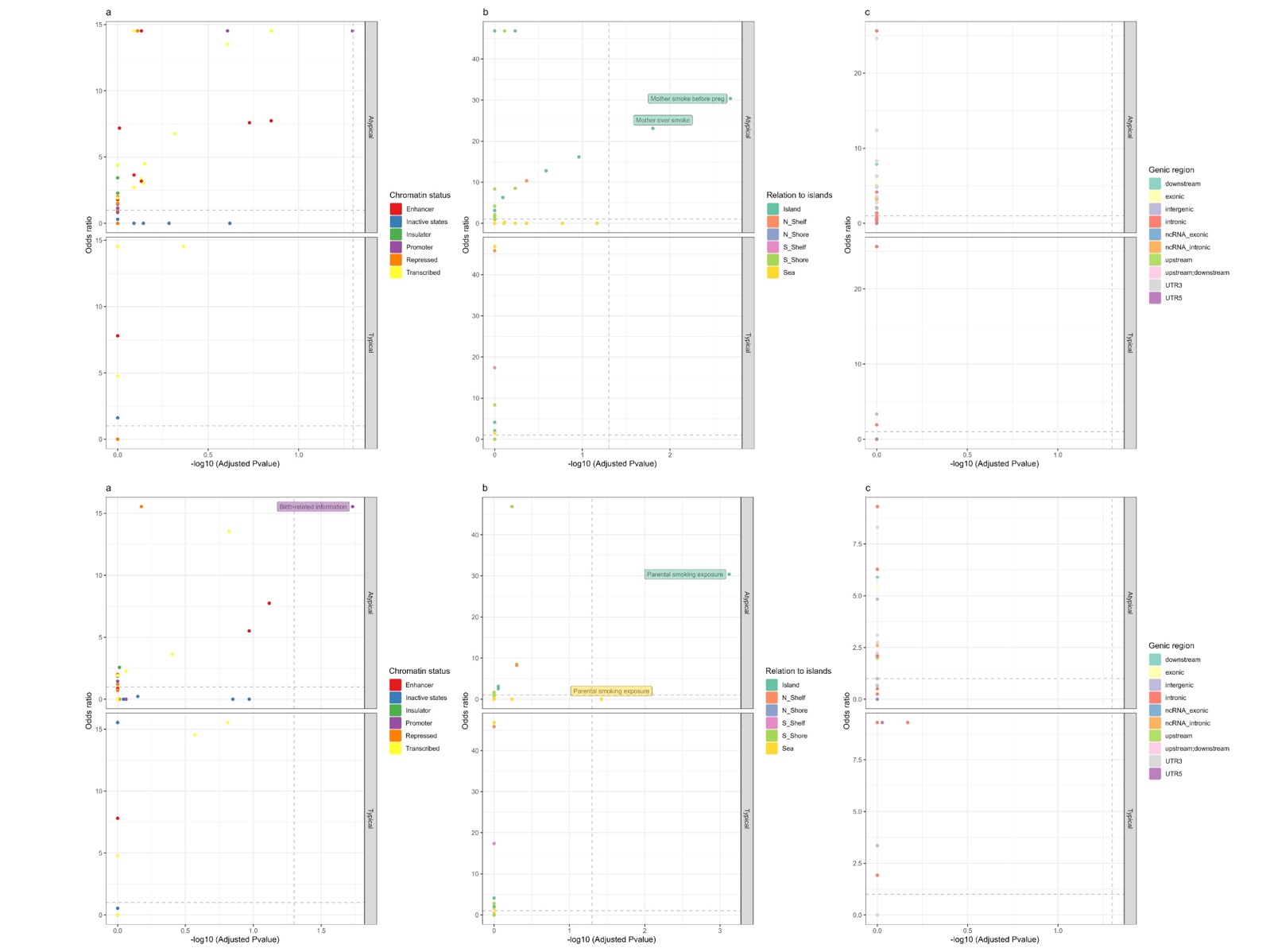


**Figure s1. Functional enrichment of associated POE-CpGs for each phenotype and each phenotypic category.** Upper figures: phenotypic level. Bottom figures: categorical level.


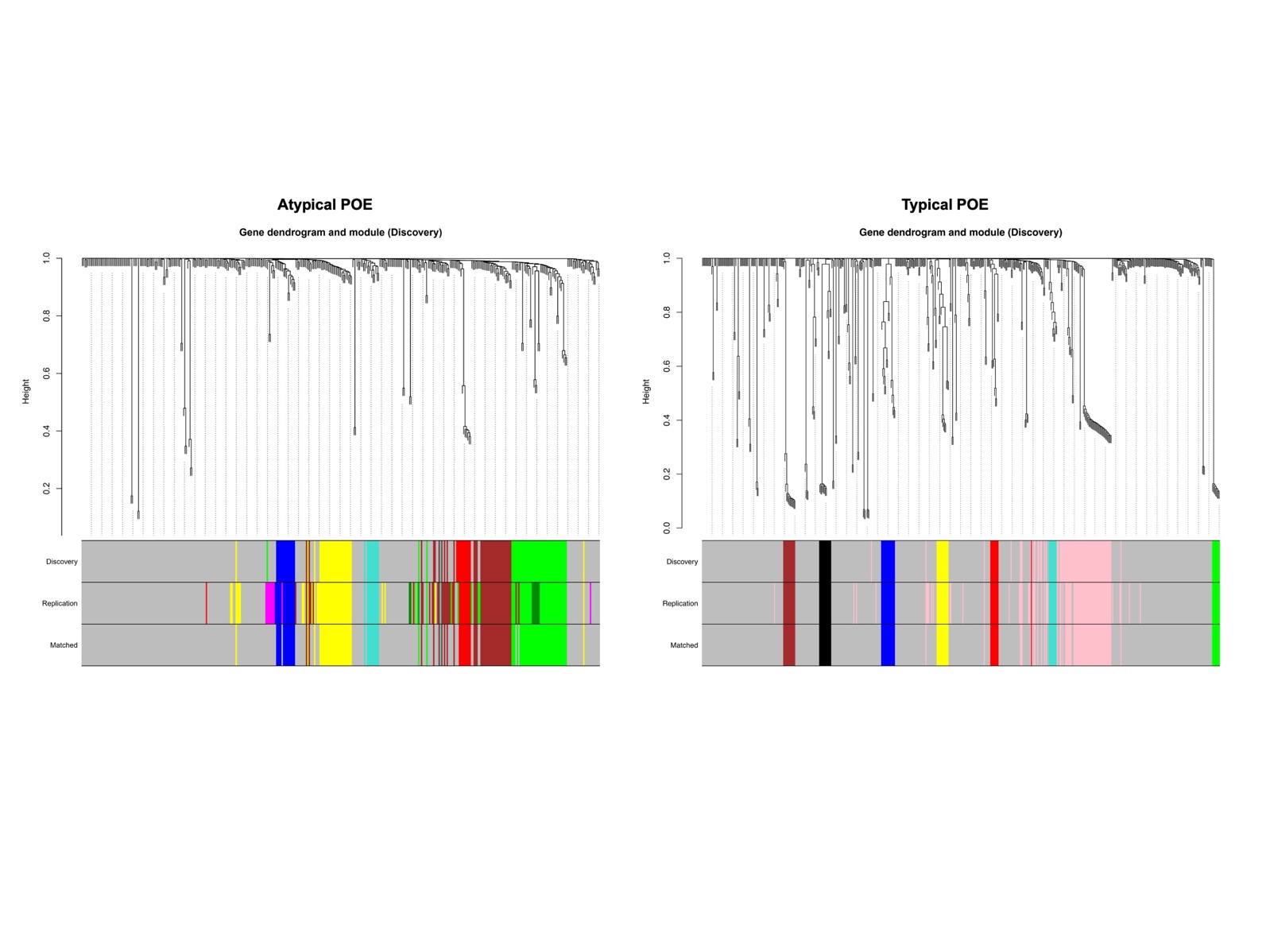


**Figure s2. Identified WGCNA POE co-methylation modules in discovery and replication datasets.**

a.


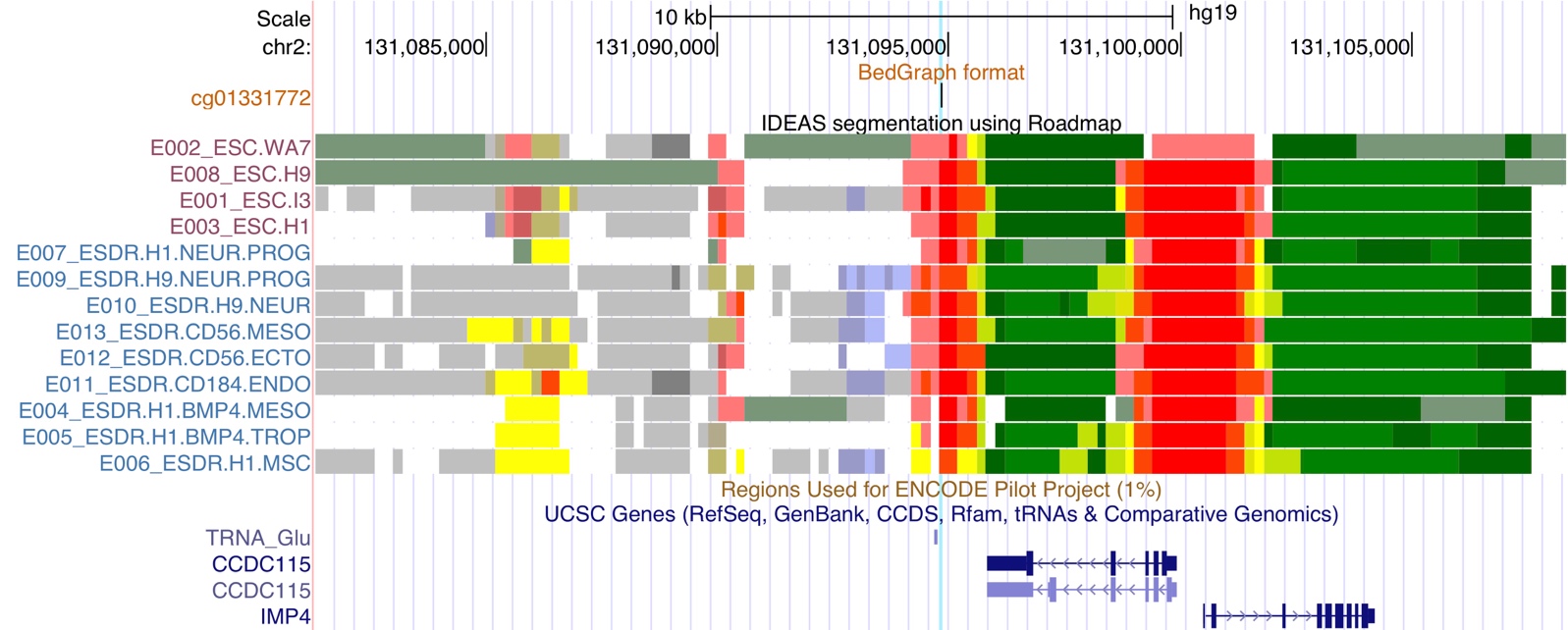


b.


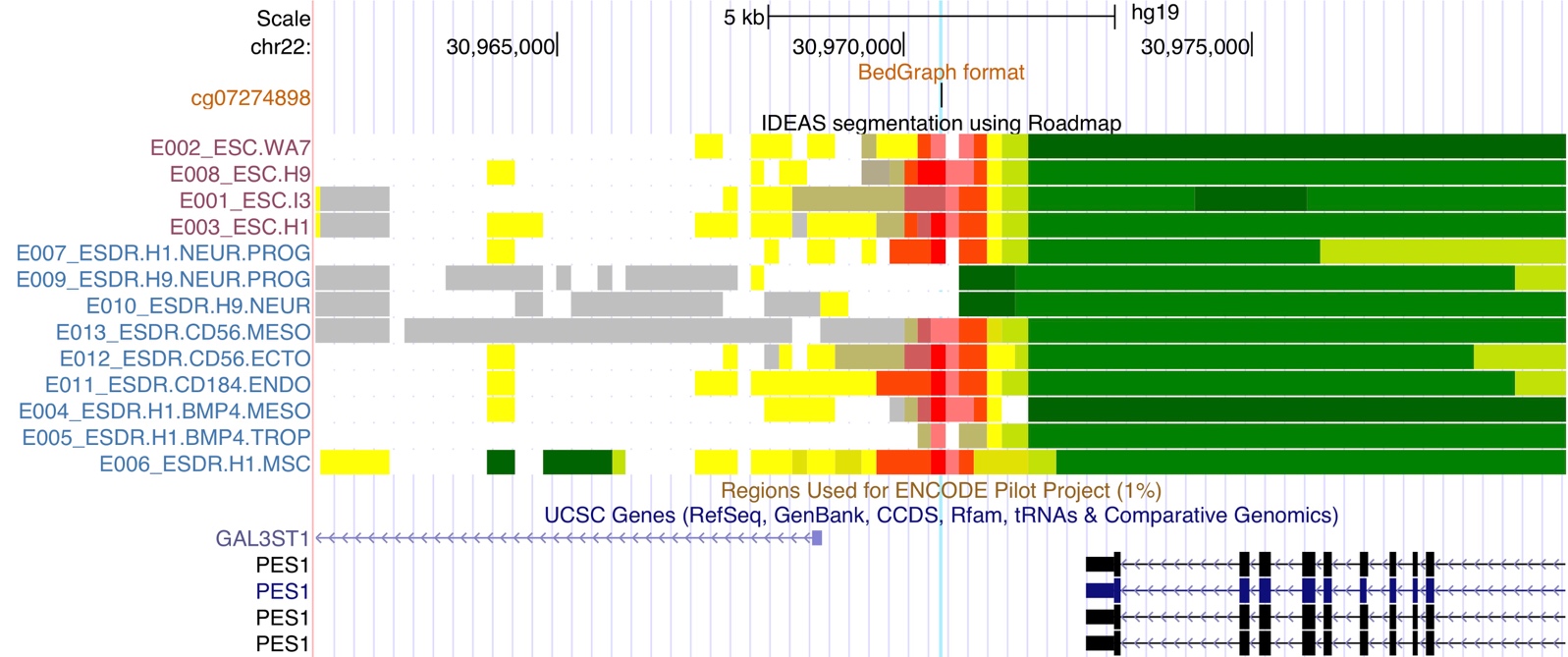


c.


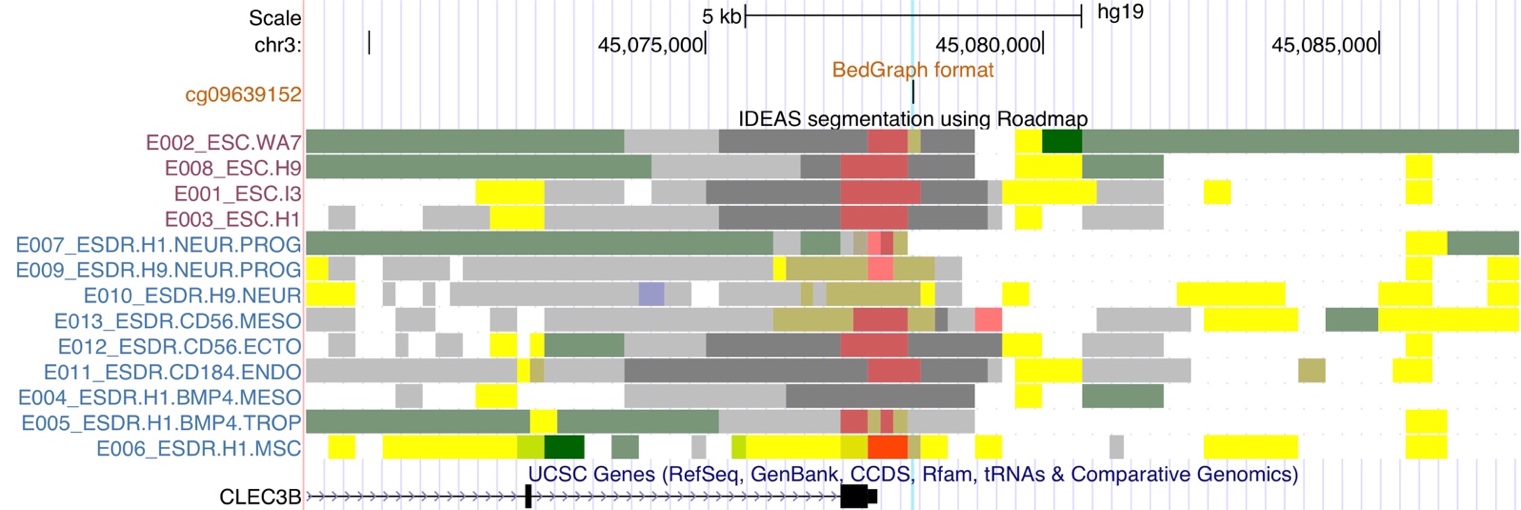


d.


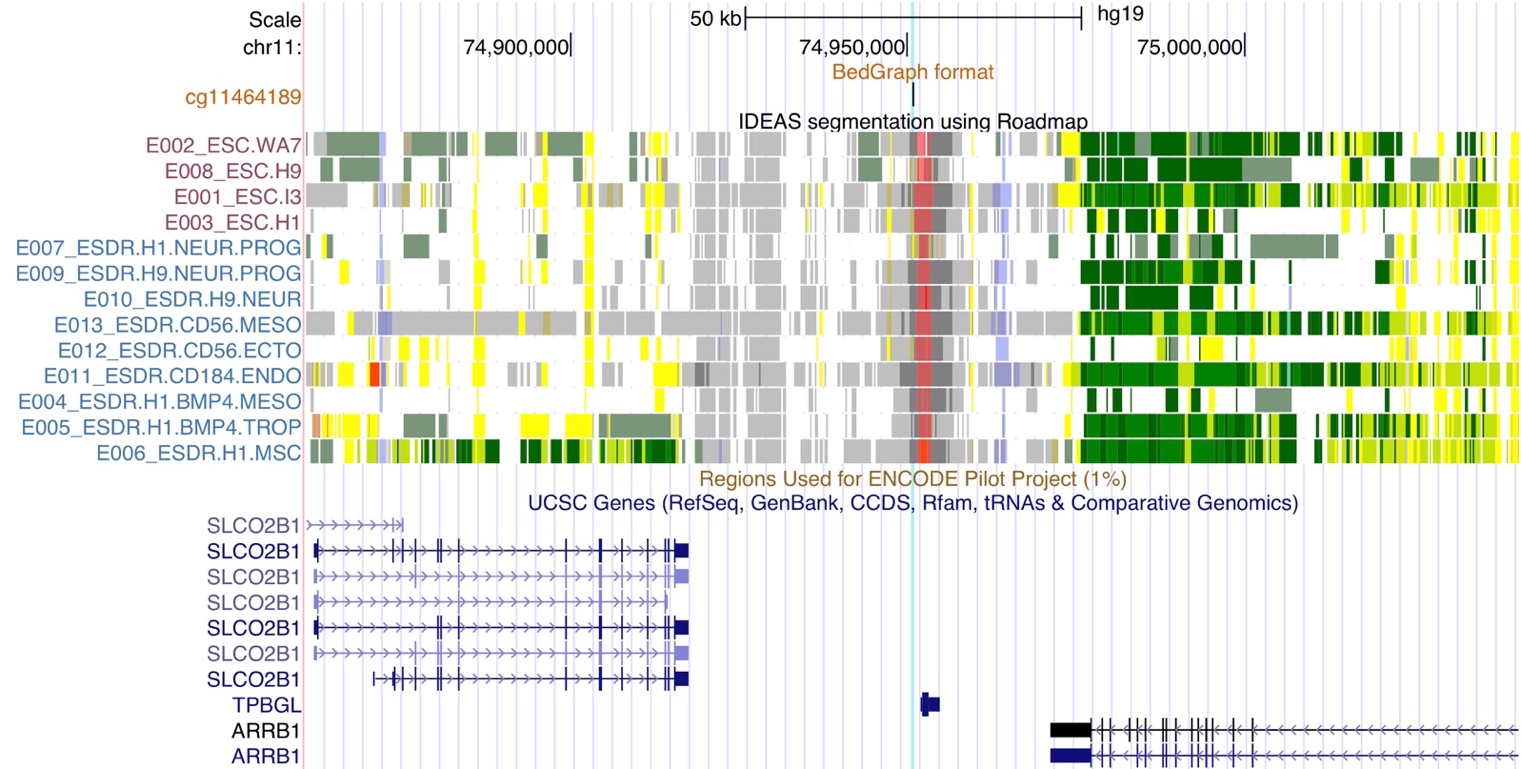


e.


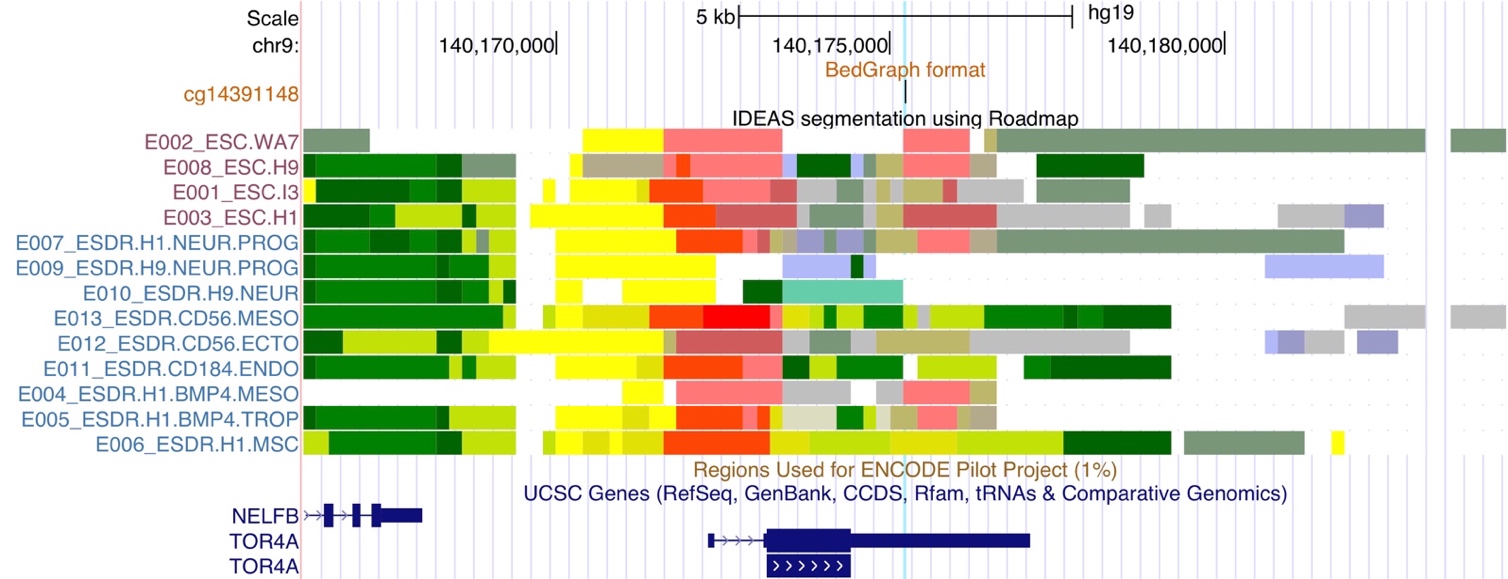


**Figure s3. Annotations for the genomic context of the five hub CpGs of the atypical POE module 3.** a: cg01331772; b: cg07274898; c: cg09639152; d: cg11464189; e: cg14391148. The color codes of IDEAS annotation using Roadmap data can be found in Figure 1a in Zhang et.al., 2017 (1).


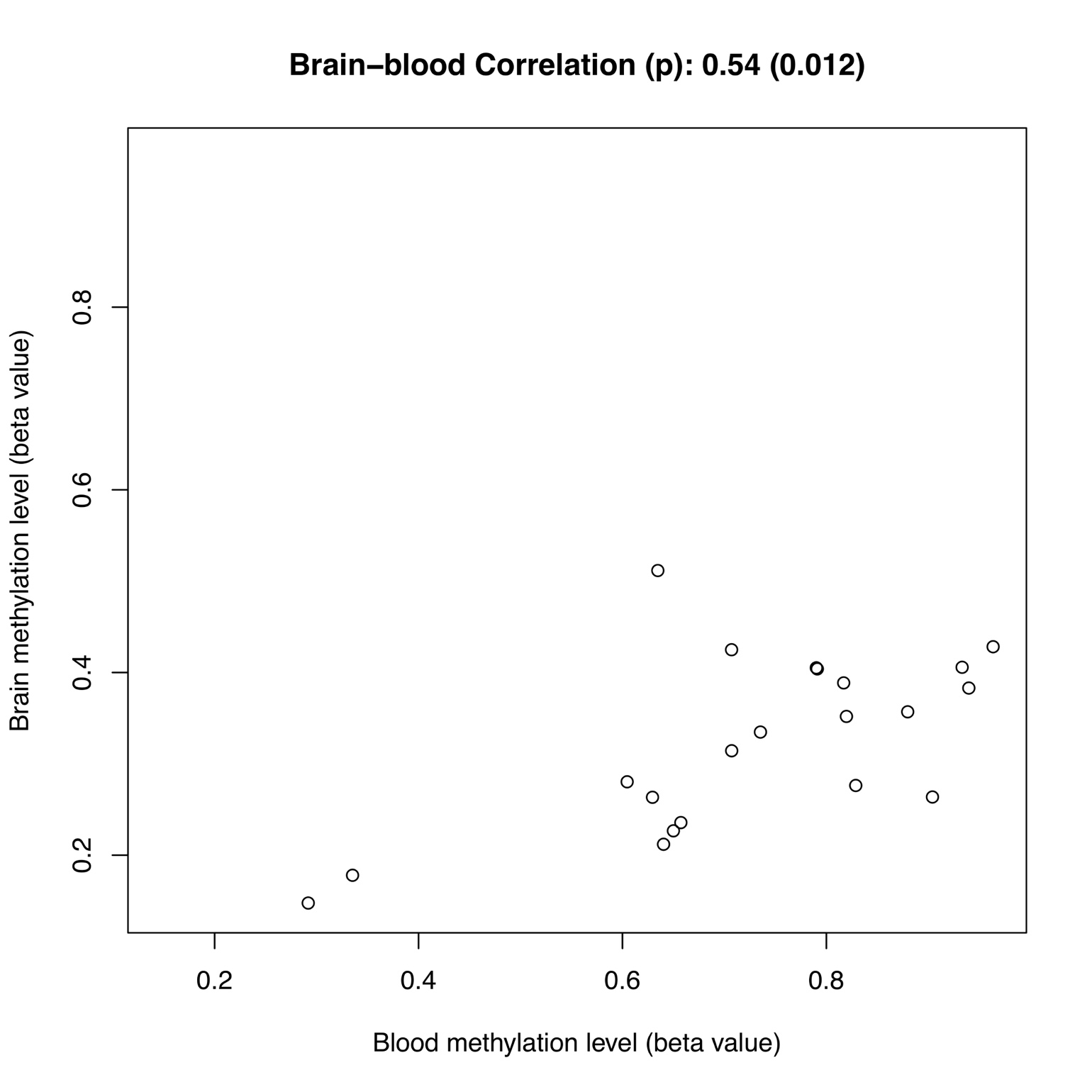


**Figure s4. The correlation of methylation levels of cg01331772 between blood and brain.** The results were extracted from IMAGE-CpG (2).


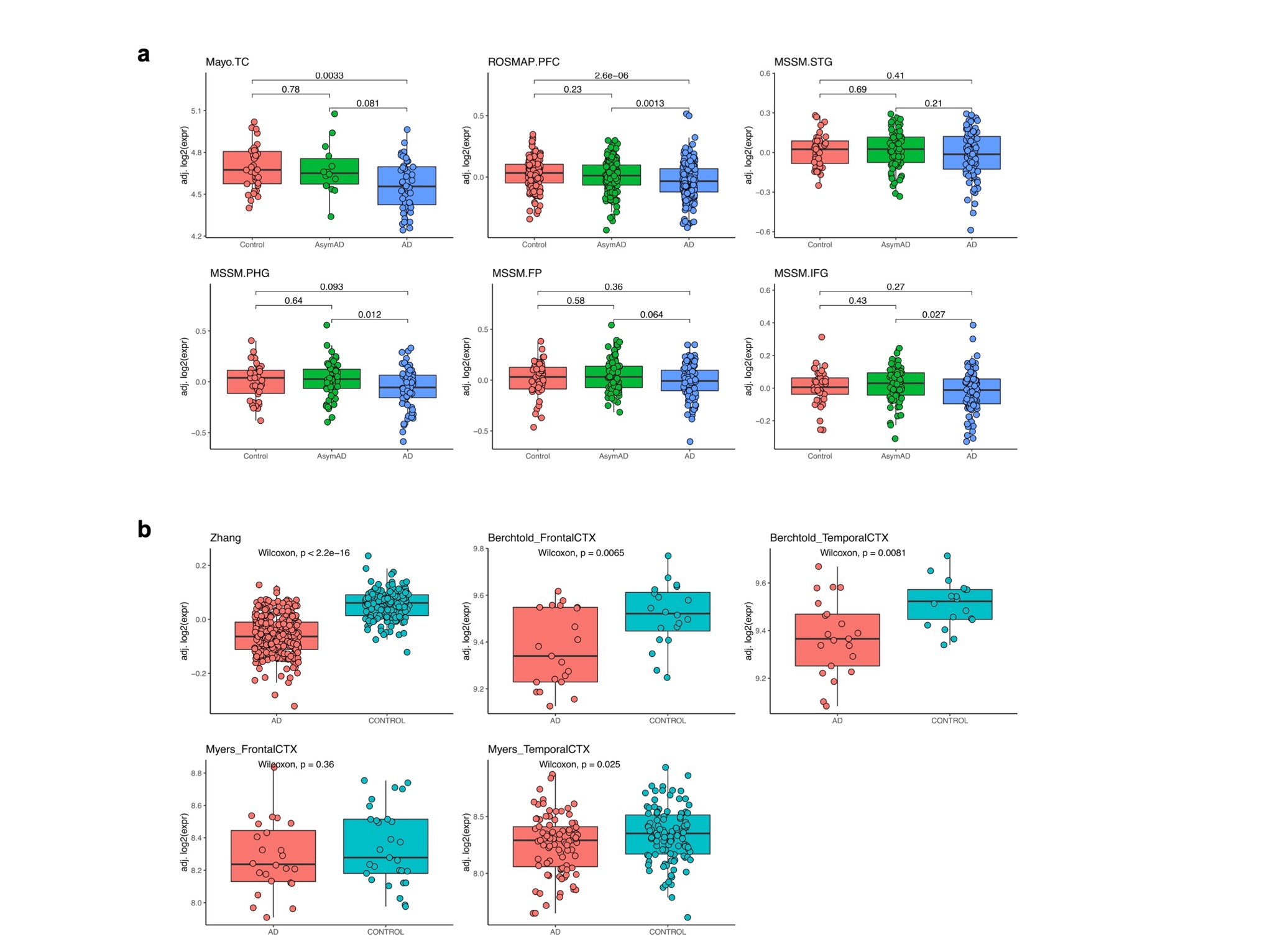


**Figure s5. Comparisons of *IMP4*'s mRNA expression in different brain tissues in control and Alzheimer's disease patients groups.** The results were extracted from http://swaruplab.bio.uci.edu:3838/bulkRNA/(3). a. Results from the consensus datasets. Mayo.TC: Mayo Clinic Brain Bank (Mayo) temporal cortex (TC); ROSMAP.PFC: Religious Orders Study and Memory and Aging Project (ROSMAP) prefrontal cortex (PFC); MSSM.STG/PHG/FP/IFG: Mount Sinai School of Medicine (MSSM) para-hippocampal gyrus (PHG), inferior frontal gyrus (IFG), superior temporal gyrus (STG) and frontal pole (FP). b. Results from the validation datasets. Zhang: prefrontal cortex in Zhang et al.(GSE44770); Berchtold_FrontalCTX/TemporalCTX: Frontal cortex and temporal cortex in PMID:23273601; Myers_FrontalCTX/TemporalCTX: Frontal cortex and temporal cortex in PMID:19361613


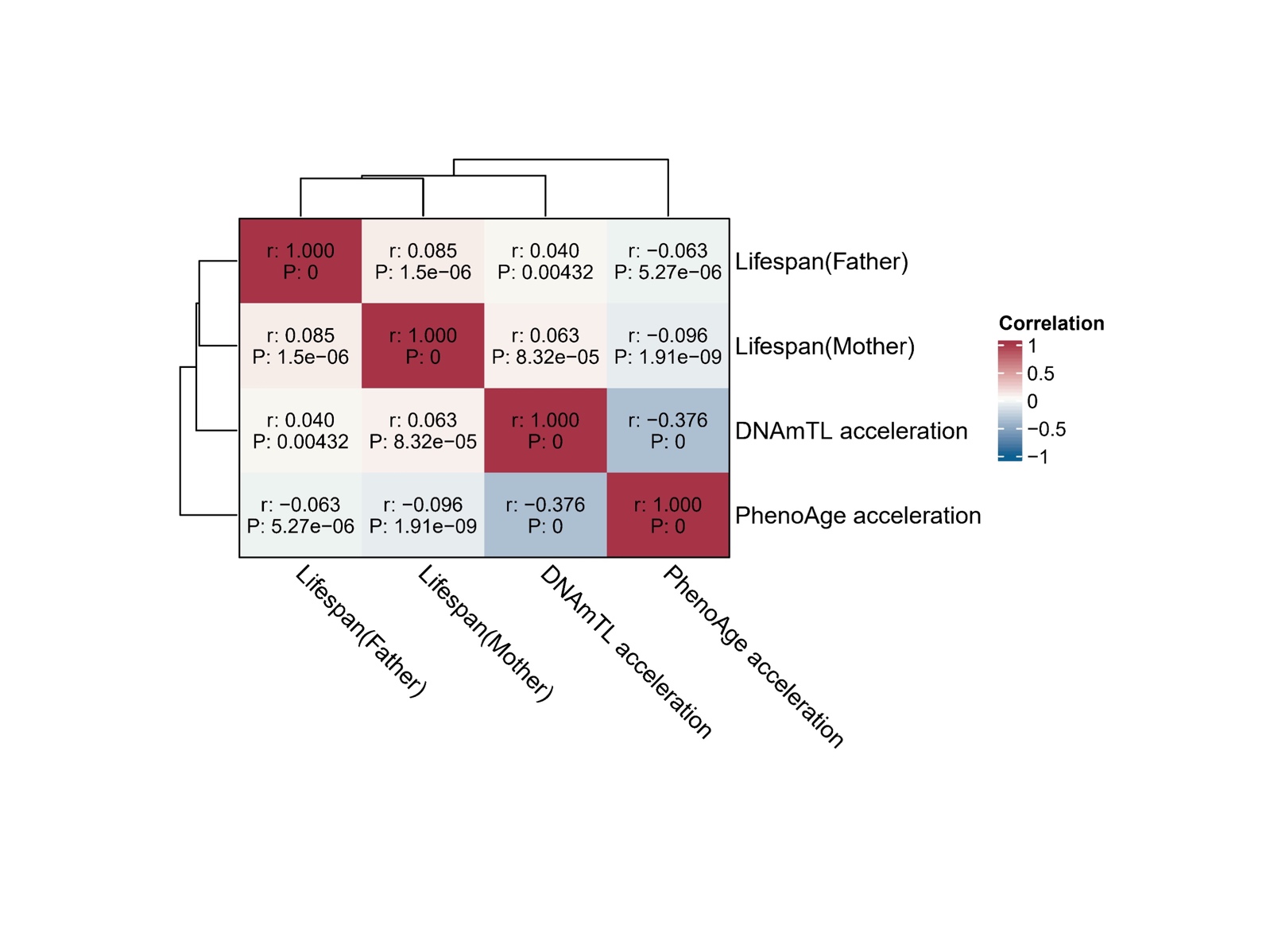


**Figure s6. Phenotypic correlations between the four aging phenotypes.**

**Reference**

1. Zhang Y, Hardison RC. Accurate and reproducible functional maps in 127 human cell types via 2D genome segmentation. Nucleic acids research. 2017;45(17):9823-36.

2. Braun P, Han S, Nagahama Y, Gaul L, Heinzman J, Hing B, et al. IMAGE-CpG: development of a web-based search tool for genome-wide DNA methylation correlation between live human brain and peripheral tissues within individuals. 2019;29:S796.

3. Morabito S, Miyoshi E, Michael N, Swarup V. Integrative genomics approach identifies conserved transcriptomic networks in Alzheimer's disease. Human molecular genetics. 2020;29(17):2899-919.
