# Supplementary material for: Phenome-wide analyses identify an association between the parent-of-origin effects dependent methylome and the rate of aging in humans
Source: Genome Biol. 2023 May 15;24:117. doi: 10.1186/s13059-023-02953-6 (PMC10184337; doi:10.1186/s13059-023-02953-6)
Supplement: Supplementary file 3 — Additional file 3. Review history. [file 13059_2023_2953_MOESM3_ESM.docx]

Review History

**First round of review**

**Reviewer 1**

**Were you able to assess all statistics in the manuscript, including the appropriateness of statistical tests used?**

Yes, and I have assessed the statistics in my report.

**Comments to author:**

This article explores associations between parent-of-origin dependent methylomes, environmental exposures at different life-stages, and aging-related phenotypes in humans. Gao et al. examined methylation at both the single CpG level as well as across co-methylation networks, providing a complementary view of how parent-of-origin effects on methylation can have functional impacts. Finally, they compare 'typical' parent-of-origin methylation imbalances related to genomic imprinting with 'atypical' imbalances that are not related and find the latter is much more sensitive to environmental factors.

The reviewer's general impression is that this study reveals novel insight into how parent-of-origin effects are influenced by environmental factors to affect aging-related and other complex traits. This paper will be of great interest to the parent-of-origin effects field but would benefit from major editing to ensure its fascinating findings are received in the most comprehensible manner. Other major comments center on expounding on the two classes of POE-CpGs analyzed, concerns over appropriate multiple test corrections, and complete figure legends. Minor comments focus on suggestions to improve readability and clarity.

Major Comments:

Editing - the syntax made it difficult to follow the analyses performed or arguments made. Sentences often contained two or three points that would be easier to comprehend if broken down into multiple, simpler, shorter sentences. The authors could also consider using the "active voice" instead of "passive voice" to report their findings more concisely and directly. Some examples of unnecessarily long, wordy, and/or complicated sentences are:

* "After accounting for the baseline number of POE-CpGs in the atypical group and that of POE-CpGs in the typical group as well as the correlation of methylation levels between POE CpGs, lifestyle and aging were the most associated phenotypic categories for POE-CpGs, in particular for the atypical POE-CpG group" (page 20)

* "Within the sharply increasing cluster (c3), five hub CpGs (cg01331772, cg09639152, cg14391148, cg07274898, cg11464189) were identified for displaying the highest centrality, connecting with the largest number of CpGs (Table s14). Also in the sharply increasing cluster (c3), the strength of methylation connectivity, in particular for connections radiating from the five hub CpGs, progressively increased during aging, with the strongest connectivity reached at the oldest (66y-94y) age group" (page 28)

o Could possibly be rewritten as: "In the sharply-increasing cluster (c3), five CpGs (X, X, X, X, X) acted as "hubs" that displayed the highest centrality and connected with the most other CpGs (Table S14). These connections also significantly increased the strength of their methylation connectivity during aging, where the oldest age group (66y-94y) had the strongest connectivity"

* "These observations not only unveiled new targets (cg01331772 and other constituent CpGs of atypical POE module 3) for future biomarker and intervention studies of aging, but also highlighted that, in order to comprehensively evaluate the multiplex role of functional CpGs such as POE-CpGs in human aging, it is necessary to consider the effects both when they act as individual sites and act as constituent members in a network, consider the dynamic association patterns in stratified age groups, and consider both the mean difference and the connectivity difference in the methylation network." (page 38)

Further, adjectives/ definite and indefinite articles to identify nouns are missing throughout the text. This is extremely distracting and should be corrected.

Typical versus atypical POE-CpGs - The comparison and contrast between these two classes of methylation patterns was one of the most interesting parts of the study. They build on a growing body of literature that describes mechanisms of parent-of-origin effects that are outside of traditional imprinting and sensitive to environmental factors. However, the weight of this distinction often felt lost throughout the paper, hidden behind jargon. It could benefit from being stressed more throughout the Results and Discussion. The classes were only vaguely defined in the Introduction and opaquely covered in the Methods.

Page 12, lines 1-3 - why and how were the 984 POE-CpGs selected? How were they classified as typical versus atypical? These CpGs are the crux of the study and its clear they build off previous work. Briefly explain in this paper why they are important and how they were identified in the previous study, instead of merely citing it. This explanation would be useful at the beginning of the Results section as well.

Methods, single CpG analyses - methylation imbalances are often correlated for CpGs within imprinted domains (see Edwards and Ferguson-Smith 2007, DOI 10.1016/j.ceb.2007.04.013). Methylation imbalances in CpGs within the same gene would also be correlated. This breaks the assumptions of independence between tests that typical false-discovery rate methods use. Of the 984 POE-CpGs selected, how many were located within the same gene and/or imprinted region? Likely several, as multiple examples are discussed in the Results section (i.e. page 25). A permutation approach may be more appropriate to correct for multiple tests, given the inherent correlation in this methylation dataset.

Figure Legends - Figure 7 has a great figure legend: the title captures the main point while the text explains the panels well-enough that the reader can understand it on its own. Consider modeling the other main figure legends based on Figure 7. Particularly, Figures 8 - 10 have no legend and need to be completed. Figures 1 - 4 and 6 have skeletal legends of incomplete sentences that are hard to understand.

Minor Comments:

Page 7, POE paragraph - Consider broadly defining POEs at the start of this paragraph before discussing their methylation-specific manifestations. The second sentence is also hard to comprehend yet introduces vital concepts for the rest of the paper. Clarify that these methylation imbalances are being determined at the across-individual level and not the allele-specific level, as genomic imprinting is an allele-specific and within-individual phenomenon. If a schematic of these patterns is included in Figure 1, it would be helpful to reference here.

Results, general comment - Genome Biology places its Methods sections at the end, so readers will encounter the Results section first. The manuscript would be greatly improved if each Results subsection began with a short summary of the "how" and "why" of the following analysis. For example, in page 20, recap the goal of the MOA and CpG-outcome models for testing associations between POE-CpG methylation and phenotypes.

Figure 1 - great conceptual summary in Panel 3. Consider reducing the amount of text and specific numbers in Panels 1 and 2, so they convey a similar high-level summary. For example, Panel 1 could include a cartoon of how typical/atypical POE patterns are defined in this study. Most text in Panel 2 could be replaced by a schematic of the single CpG level and module level analyses performed, where the bolded results are highlighted. A logo or graphic for the GS:SFHS study would also fit nicely in Panel 1.

Figure 3 - the plot text is too tiny to be readable, especially for the bar charts. The panels could be reformatted to better use the empty white space on the page (i.e. become wider).

Figure 5 - the figure legend should indicate that POE-CpGs located within the same gene are grouped together by a dotted black oval. Do the differences in gene name color (purple or green) mean anything? Also, thicker line widths between nodes would improve readability; the dotted red lines between two CpGs and aging are hard to detect.

Figure 6 - increase font size of the phenotype labels and facet panels (bottom and top x-axis)

Pages 12-15, "pre-correction" - it seems the single POE-CpG methylation levels were "pre-corrected" for several variables, then the same adjusted values were used in multiple analyses. If true, then those data correction steps could be described once in their own section (located after "Phenotype Data" section) instead of similar text being repeated in each analysis section (MOA models through WGCNA networks).

Page 13 - two sentences about whether variables were pre-adjusted are repeated twice in the same paragraph. Remove one instance.

Page 14, CpG-Outcome Model section - what were the statistical significance thresholds? Any multiple testing corrections performed? If yes, how so?

Pages 13-14, MOA model - what were the FDR-corrected statistical significance thresholds?

Pages 16-17, PWAS modules - were any multiple testing corrections performed? If yes, how so and what were the statistical significance thresholds?

Page 28, first paragraph - specify the age groups in the main text. Consider including the specific mean connectivities of the youngest and oldest groups as an example comparison.

Acronyms, general comment - define acronyms before their first use to aide unfamiliar readers. For example, write out the full GS:SFHS study name at its first mention in the Introduction (page 9, lines 1-2) and in the Population Samples subsection of the Methods (page 10). The study could also be fully spelled out in Figure 1. Also, "PCs" on page 27, as that will be its first use.

**Reviewer 2**

**Were you able to assess all statistics in the manuscript, including the appropriateness of statistical tests used?**

Yes, and I have assessed the statistics in my report.

**Comments to author:**

Gao, et al. present a very strong and comprehensive analysis of an important emerging topic, parent-of-origin effects in DNA methylation and the association of methylation at the CpG loci with various aging-related traits. One issue with the data, as presented, is that the analysis is of methylation sites previously demonstrated to have differential methylation profiles depending on the parent-of-origin. Thus, the analysis itself does not truly speak to differences in long-term health based on the maternal vs paternal origin of methylation changes, but rather informs on the extent to which such POE-dependent sites influence long-term health and disease. This isn't a major problem, but the title and abstract could make the true nature of the analysis and results clearer. Some further concerns could be addressed to strengthen what is, overall, a very strong manuscript.

Introduction:
1) "Biological aging explained by DNA sequences, however, only accounts for influences from predisposing and unchangeable risk factors". This is specific to "heritable/constitutive DNA variation", as somatic DNA changes obviously influence biological aging. Mosaic loss of entire chromosomes are one such example outside the context of neoplastic disease.
2) "When it comes to early effects, a few studies reported association between early exposures and age acceleration in newborns and children, but not in adults (not tested)(13, 14)." Although an exhaustive list of such studies is unnecessary, it may be valuable to cite somewhere the emerging analyses of age-acceleration in newborns with Trisomy 21 (eg, PMID: 35661546).
3) "To date, only one human study reported the association between methylation levels of POE-influenced genes and the change of brain structures overtime". "Overtime" should be two words.

Methods:
4) "The list of 984 POE-CpGs (Natypical=398, Ntypical=586) was extracted from a previous study(18)." This study was from these authors apparently using the same samples as the current Discovery set. There's nothing intrinsically wrong with such a design, but it should be made clear that the datasets nearly entirely overlap and acknowledge any caveats in reproducibility that this might entail.

Results:
5) "The phenotypic categories contributed the largest number of associations were parental smoking exposure, lifestyle, intelligence and aging (Figure 3a)." "Contributed" should be "contributing".
6) "Conditional analyses suggested that the associations with parental smoking were mainly driven by maternal smoking". Is it possible to do these as purely stratified analyses? Or was paternal smoking data too sparse?
7) "Conditional analyses indicated that the multiple associations of these POE-CpGs were not driven by the socioeconomic status (measured as SIMD)(Table s8)." This should be expanded using methylation as outcome and modeling all phenotypes together to assess whether smoking (or even IQ) is mediating the other phenotypic associations.

Discussion:
8) Some discussion of the potential for genetic confounding to play a role in amplifying results seems merited. How much is maternal/paternal IQ influencing parental behavior and child exposures, and playing out here as methylation-associations with subject intelligence (when just mediated by classical genetic SNP-based associations)?

**Authors Response**

**Point-by-point responses to the reviewers’ comments:**

We thank the reviewers for the very constructive comments, they have certainly contributed to the improvement of this manuscript. Below we show reviewers’ comments **in *Bold Italic*** and our responses in plain text. **The page numbers** mentioned below are from the **revised manuscript (changes tracked version.** The page number is printed in the **bottom right** of each page).

***Reviewer #1: This article explores associations between parent-of-origin dependent methylomes, environmental exposures at different life-stages, and aging-related phenotypes in humans. Gao et al. examined methylation at both the single CpG level as well as across co-methylation networks, providing a complementary view of how parent-of-origin effects on methylation can have functional impacts. Finally, they compare 'typical' parent-of-origin methylation imbalances related to genomic imprinting with 'atypical' imbalances that are not related and find the latter is much more sensitive to environmental factors.***

***The reviewer's general impression is that this study reveals novel insight into how parent-of-origin effects are influenced by environmental factors to affect aging-related and other complex traits. This paper will be of great interest to the parent-of-origin effects field but would benefit from major editing to ensure its fascinating findings are received in the most comprehensible manner. Other major comments center on*** ***expounding on the two classes of POE-CpGs analyzed, concerns over appropriate multiple test corrections, and complete figure legends. Minor comments focus on suggestions to improve readability and clarity.***

We thank the reviewer for these encouraging comments. We now carefully address the reviewer’s comments as below.

***Major Comments:***

***Q1: Editing - the syntax made it difficult to follow the analyses performed or arguments made. Sentences often contained two or three points that would be easier to comprehend if broken down into multiple, simpler, shorter sentences. The authors could also consider using the "active voice" instead of "passive voice" to report their findings more concisely and directly.***

A1: we apologize that the previous version of the paper was not concise and direct in a number of places. In revised version, we made several changes to improve the readability (for detailed changes, please, see the answers below)

***Q1-1: Some examples of unnecessarily long, wordy, and/or complicated sentences are:***

****	"After accounting for the baseline number of POE-CpGs in the atypical group and that of POE-CpGs in the typical group as well as the correlation of methylation levels between POE CpGs, lifestyle and aging were the most associated phenotypic categories for POE-CpGs, in particular for the atypical POE-CpG group" (page 20)***

A1-1: This has been revised in the page 10 of the new manuscript. It now reads as:

“We next ranked the phenotypic categories by the normalized counts of associated POE-CpGs after accounting for the baseline numbers of POE-CpGs in the atypical and the typical group respectively, as well as the correlation of methylation levels among POE-CpGs (Figure 3b). This revealed that lifestyle and aging were the most associated phenotypic categories for POE-CpGs, in particular for the atypical group (Figure 3b). Smoking status and DNAmTL acceleration were the most associated phenotypes (Figure 3c).”

***Q1-2: *	"Within the sharply increasing cluster (c3), five hub CpGs (cg01331772, cg09639152, cg14391148, cg07274898, cg11464189) were identified for displaying the highest centrality, connecting with the largest number of CpGs (Table s14). Also in the sharply increasing cluster (c3), the strength of methylation connectivity, in particular for connections radiating from the five hub CpGs, progressively increased during aging, with the strongest connectivity reached at the oldest (66y-94y) age group" (page 28)***

***o	Could possibly be rewritten as: "In the sharply-increasing cluster (c3), five CpGs (X, X, X, X, X) acted as "hubs" that displayed the highest centrality and connected with the most other CpGs (Table S14). These connections also significantly increased the strength of their methylation connectivity during aging, where the oldest age group (66y-94y) had the strongest connectivity"***

A1-2: This has been revised in the page 23 of the new manuscript. It now reads as:

“In the sharply-increasing cluster (c3), five CpGs (cg01331772, cg09639152, cg14391148, cg07274898, cg11464189) acted as the "hubs" that displayed the highest centrality, connected with the most other CpGs (Table s12). The methylation connections radiated from the five hub POE-CpGs significantly increased the strength during aging, with the strongest connectivity detected in the oldest age group (66y-94y) (Figure 7c, Table s13).”

***Q1-3:*	"These observations not only unveiled new targets (cg01331772 and other constituent CpGs of atypical POE module 3) for future biomarker and intervention studies of aging, but also highlighted that, in order to comprehensively evaluate the multiplex role of functional CpGs such as POE-CpGs in human aging, it is necessary to consider the effects both when they act as individual sites and act as constituent members in a network, consider the dynamic association patterns in stratified age groups, and consider both the mean difference and the connectivity difference in the methylation network." (page 38)***

A1-3: This has been revised in the page 33 of the new manuscript. It now reads as:

“These observations unveiled new targets (cg01331772 and other constituent CpGs of the atypical POE module 3) for future biomarker and intervention studies of aging. They also highlighted that, in order to comprehensively evaluate the multiplex role of functional CpGs such as POE-CpGs in the human aging, it is necessary to consider the effects both when CpGs act as individual sites and act as constituent members in a network. The aging-association patterns appear to be dynamic in different age groups for at least some functional CpGs. The mean methylation levels and the connectivity strength could reveal different aspects of the methylation network.”

***Q2: Further, adjectives/ definite and indefinite articles to identify nouns are missing throughout the text. This is extremely distracting and should be corrected.***

A2: we apologize for this. we now have added the missed adjectives/ definite and indefinite articles in revised paper.

***Q3: Typical versus atypical POE-CpGs - The comparison and contrast between these two classes of methylation patterns was one of the most interesting parts of the study. They build on a growing body of literature that describes mechanisms of parent-of-origin effects that are outside of traditional imprinting and sensitive to environmental factors. However, the weight of this distinction often felt lost throughout the paper, hidden behind jargon. It could benefit from being stressed more throughout the Results and Discussion. The classes were only vaguely defined in the Introduction and opaquely covered in the Methods***.

A3: We now have more comprehensively introduced the classes and increased the discussion about the comparisons of the two subgroups in the Results and Discussion. An example is the added discussion in page 34 of the new manuscript:

“This study revealed substantial differences between the POE CpGs belonging to the atypical and typical groups. Imprinting-related POE (the typical type) have been previously associated with metabolic, behavioral, and neurological traits (1). Although our results also supported those associations (Figure 3), we found that the methylation associations with those traits are not enriched in the typical POE regions (Figure 4), indicating the complex mechanisms of those traits and the relatively small overall influence of imprinting for those traits at the population level. Compared to the relatively well studied imprinting-related POE methylome (the typical type), the atypical POE-influenced methylome has been largely understudied. Our study provided multi-level evidence for the environment-sensitive and aging-related features of the atypical POE-influenced methylome. The early effects from maternal smoking and maternal age when the child was born on DNA methylation were found to be highly enriched in those regions (Figure 4). The strong associations with adult aging and other health phenotypes further revealed the dynamic features of the atypical POE-CpGs throughout the life. These results emphasized the need for future research on this sensitive and flexible part of the methylome.”

***Q4: Page 12, lines 1-3 - why and how were the 984 POE-CpGs selected? How were they classified as typical versus atypical? These CpGs are the crux of the study and its clear they build off previous work. Briefly explain in this paper why they are important and how they were identified in the previous study, instead of merely citing it. This explanation would be useful at the beginning of the Results section as well.***

A4: the explanation has now been added at the beginning of the results section (page 9 in the new manuscript).

“The list of 984 POE-CpGs was extracted from Zeng et.al 2019(2). Those CpGs displayed imbalanced methylation similarity between mother-offspring, father-offspring and siblings (Figure 1), and were identified using overlapping samples with the current discovery dataset (2). Among them, 943 POE-CpGs passed the quality control for the DNA methylation data in this study. Below, we displayed the results from the analyses performed separately for the POE-CpGs belonging to the atypical and typical subgroups. The distinct features of these subgroups have been revealed previously: the typical POE-CpGs (N_QCed_typical_=560) are strongly enriched for imprinted regions and have POE-mQTLs detected, whereas the atypical POE CpGs(N_QCed_typical_=383) are not enriched for imprinted regions and have no POE-mQTLs detected (Figure 1) (2).”

***Q5: Methods, single CpG analyses - methylation imbalances are often correlated for CpGs within imprinted domains (see Edwards and Ferguson-Smith 2007, DOI 10.1016/j.ceb.2007.04.013). Methylation imbalances in CpGs within the same gene would also be correlated. This breaks the assumptions of independence between tests that typical false-discovery rate methods use. Of the 984 POE-CpGs selected, how many were located within the same gene and/or imprinted region? Likely several, as multiple examples are discussed in the Results section (i.e. page 25). A permutation approach may be more appropriate to correct for multiple tests, given the inherent correlation in this methylation dataset.***

A5: Among the 984 POE-CpGs, 596 were located within 2Mb (234 located within 2Kb) of known imprinted regions. It is true that the methylation levels of some POE-CpGs are correlated. In the previous paper (Zeng et.al., 2019)(2), a clumping procedure that only keeps one index POE-CpG for each correlated POE-CpG cluster (R^2^>0.1) leads to 733 independent index POE-CpGs. As can be seen in additional file 2, the CpGs belonging to the same genes tend to be correlated and for those genes usually 1-2 index POE-CpGs were identified per gene.

We agree with the reviewer that for the data with a correlated structure, permutation-based multiple testing correction would the best to account for structure. However, in our current analyses, it would be quite computationally challenging to perform a large enough number of permutations for a linear mixed model method (MOA) in ~5000 samples (discovery dataset). In fact, the FDR method has been proven to also control the false discovery rate well when the tests are not independent(3). For this reason, the FDR method has been commonly used in studies of correlated features such as RNA-seq data (4). This is the reason why we used the FDR method in the single-CpG based tests. It is also important to note that the MOA is already a relatively conservative method that controls for the correlation between probes by fitting the Omic relationship matrix as a random effect(5). In our results, the vast majority of the significant results produced by using MOA at the discovery stage were also well replicated using the same method on independent samples. Additionally, they were well validated by the classical CpG-outcome model in both datasets (94%).

To be conservative, we only reported the results that are replicated and validated as “high confidence results” and only discussed those results in the paper. We acknowledge that the results are only tested in the Scottish population, and we are aiming to extend the replication of these results to different populations, as discussed in the limitations in page 34-35 of the new manuscript.

***Q6: Figure Legends - Figure 7 has a great figure legend: the title captures the main point while the text explains the panels well-enough that the reader can understand it on its own. Consider modeling the other main figure legends based on Figure 7. Particularly, Figures 8 - 10 have no legend and need to be completed. Figures 1 - 4 and 6 have skeletal legends of incomplete sentences that are hard to understand.***

A6: we apologize for that. We now have completed the legend of Figure 8-10 and edited the sentences in the legends of other figures.

***Minor Comments:***

***Q7-1: Page 7, POE paragraph - Consider broadly defining POEs at the start of this paragraph before discussing their methylation-specific manifestations. The second sentence is also hard to comprehend yet introduces vital concepts for the rest of the paper. Clarify that these methylation imbalances are being determined at the across-individual level and not the allele-specific level, as genomic imprinting is an allele-specific and within-individual phenomenon.***

A7-1: we have revised these sentences in the POE paragraph as the following (page 5 of the new manuscript):

“For a DNA methylation site, the POE at the individual level was traditionally defined as any genetic effect of magnitude dependent on the parent-of-origin inheritance of alleles(6). At the population level, we previously reported that the POE-influenced methylome manifested imbalanced methylation similarity between nuclear family members of the same genetic distance (mother-offspring, father-offspring, sibling-sibling pairs) (Figure 1)(2). The CpG sites displaying the imbalanced methylation pattern could be further divided into two groups, the typical and the atypical types (Figure 1). The typical POE-CpGs are the group for which specific regulatory SNPs (POE-mQTLs) have been identified for influencing the methylation levels of the target CpGs through introducing the parent-of-origin-dependent SNP effect (2, 7). This type of POE-CpGs is highly enriched in the regions targeted by genomic imprinting, a biological process happening at early developmental stages and the resulted epigenetic status needed to be well maintained/regulated throughout the life (7). The epigenomic features influenced by the typical POEs have been found to be sensitive to prenatal and postnatal environmental stimuli, such as maternal nutrition during pregnancy and stress accompanied with assisted reproductive technologies(8-12). In contrast, some methylation sites also display the imbalanced methylation patterns but have no identified POE-mQTLs, and are not enriched in known imprinted regions. These sites should, therefore, be regarded as “atypical POE-CpGs”(2). Since dominance genetic effects have been ruled out for the majority of these atypical POE-CpGs(2), potential explanations for the atypical POE pattern are either small POE-mQTL (imprinting) effect not yet detected due to the lack of power, or early familial environmental effects introduced by the parents(2). In any case, both typical and atypical POE-CpGs represent classes of CpGs for which methylation levels are heavily influenced by early life events.”

***Q7-2: If a schematic of these patterns is included in Figure 1, it would be helpful to reference here.***

A7-2: we have added the schematic of the patterns in the new Figure 1 and referenced that in the sentence (as answered in A7-1).

***Q8: Results, general comment - Genome Biology places its Methods sections at the end, so readers will encounter the Results section first. The manuscript would be greatly improved if each Results subsection began with a short summary of the "how" and "why" of the following analysis.***

A8: We have moved the Methods to the space after the Discussion. We have made changes to make sure the beginning sentences of each sub-section in the results briefly introduce the purpose and the analyses of the following results.

***Q8-1: For example, in page 20, recap the goal of the MOA and CpG-outcome models for testing associations between POE-CpG methylation and phenotypes.***

A8-1: The following sentences have been added at the beginning of the single-CpG section (new manuscript: page 9):

“To identify the association between the methylation levels at each POE-CpG site and each phenotype, we applied the Mix-linear-model-based Omic Association (MOA)(5), a linear mixed model method that adjusts for the global correlation between probes, to account for unobserved confounders in the phenome-wide scan (N_CpG_=934, N_phenotype_=142) and the replication of significant results (methods). For the replicated results, we additionally used the CpG-outcome model, a classical linear regression model which avoids pre-adjustment of methylation-related variables to further validate the robustness of the MOA results (see methods section).

”

***Q9: Figure 1 - great conceptual summary in Panel 3. Consider reducing the amount of text and specific numbers in Panels 1 and 2, so they convey a similar high-level summary. For example, Panel 1 could include a cartoon of how typical/atypical POE patterns are defined in this study. Most text in Panel 2 could be replaced by a schematic of the single CpG level and module level analyses performed, where the bolded results are highlighted. A logo or graphic for the GS:SFHS study would also fit nicely in Panel 1.***

A9: we have made these changes in the new manuscript of Figure 1 (below). One thing to note is that the number of replicated associations in module-based association is 26, not the 25 in our previous version, as we identified an error during this revision (we apologize for this!). It is now corrected and none of the main results/conclusion are influenced.

***Q10: Figure 3 - the plot text is too tiny to be readable, especially for the bar charts. The panels could be reformatted to better use the empty white space on the page (i.e. become wider).***

A10: we have made these changes in the new manuscript of Figure 3 (below).

***Q11: Figure 5 - the figure legend should indicate that POE-CpGs located within the same gene are grouped together by a dotted black oval. Do the differences in gene name color (purple or green) mean anything? Also, thicker line widths between nodes would improve readability; the dotted red lines between two CpGs and aging are hard to detect.***

A11: The gene names displayed are those mentioned in the main text. The gene names with purple background: the POE-CpGs within that gene were associated with phenotypes from multiple trait categories. As suggested, we have improved the legends and updated the figure (below).

***Q12: Figure 6 - increase font size of the phenotype labels and facet panels (bottom and top x-axis)***

A12: we have made these changes in the new manuscript of Figure 6 (below).

***Q13: Pages 12-15, "pre-correction" - it seems the single POE-CpG methylation levels were "pre-corrected" for several variables, then the same adjusted values were used in multiple analyses. If true, then those data correction steps could be described once in their own section (located after "Phenotype Data" section) instead of similar text being repeated in each analysis section (MOA models through WGCNA networks)***

A13: In the single-CpG based tests, the covariate sets used to pre-correct the methylation levels are not exactly the same for the MOA and CpG-outcome models. This was because the former takes methylation as predictor and the later takes methylation as outcome. To account for the genetic structure in the discovery cohort, the outcome variable needs to be pre-corrected for the random effects represented in genomic relationship matrices, whereas the predictor does not need to be. In addition, the CpG-outcome model allows the joint fitting of methylation related factors (such as smoking, cell counts) (page 39 in the new manuscript) rather than pre-correcting them from the outcome variable. Therefore, to avoid the readers being confused by the covariates used in each analysis, we listed them in each section. For the unnecessarily duplicated descriptions in the same section, we now have removed the duplicated one (please see the answers to Q14).

***Q14: Page 13 - two sentences about whether variables were pre-adjusted are repeated twice in the same paragraph. Remove one instance.***

A14: the repeated one was removed in the new manuscript (page 38 in the new manuscript).

***Q15: Page 14, CpG-Outcome Model section - what were the statistical significance thresholds? Any multiple testing corrections performed? If yes, how so?***

A15: The FDR method was used to correct for the multiple testing here. Since the CpG-outcome model was used to validate the significance results reported by the MOA model, we only tested the CpG-outcome model for the replicated results reported by the MOA model (N=98). So the number of tests to be corrected by FDR is 98 for the CpG-outcome model in the discovery sample. The results revealed that 97 of the 98 association pairs are significant after FDR correction (P_adjust_<0.05, the highest original P value=0.009). We then tested the CpG-outcome model for the 97 pairs using the replication samples (N_test_=97). The results from that were corrected by FDR too, leading to 92 significant pairs. The step-by-step test P values and FDR corrected P values were listed in Table s1. We now have added descriptions of the multiple testing correction method and the number of tests to correct for in page 39 in the new manuscript.

***Q16: Pages 13-14, MOA model - what were the FDR-corrected statistical significance thresholds?***

A16: The number of test in the discovery stage is 934*142=132628, the number of test in the replication stage is N_tests_in_replication_=N_significant_pairs_in_discovery_=115. The FDR was applied to both the discovery and replication stages. At the discovery stage, 115 POE-CpG-phenotype pairs are significant at the FDR 0.05 level, the highest P value among the significant results is 4.3x10^-5^. At the replication stage, 98 pairs are significant at the FDR 0.05 level, the highest P value among the significant results is 0.04. We now have added descriptions of the multiple testing method and the number of tests to correct for in page 38 in the new manuscript.

***Q17: Pages 16-17, PWAS modules - were any multiple testing corrections performed? If yes, how so and what were the statistical significance thresholds?***

A17: In the module-based association tests, the Bonferroni method was used to correct for the multiple testing. The reason that we used a more stringent correction rather than using the FDR as in the single-CpG based test is because in the modular test, it is not appropriate to use the conservative MOA method because the MOA method jointly fits the Omic relationship matrix to remove the effects from the correlations between CpGs, whereas the fundamental theory of the module-phenotype associations is to test the association between the phenotype and the shared variation of CpGs within the module. Therefore, a simple linear fixed regression is more appropriate for the module-based association tests, however it might increase the chance of false positives. Hence we opted for the use of a more conservative multiple test correction and we applied the Bonferroni method in the modular associations tests in both discovery and replication samples. At the discovery stage (N_test_=3199), the Bonferroni 0.05 threshold for significance is 1.6x10^-5^_,_ there are 35 significant PC-phenotype pairs. At the replication stage, N_test_=35, the Bonferroni 0.05 threshold is 0.001, there are 26 significant pairs. We now have added descriptions for the multiple testing in page 42 in the new manuscript.

***Q18: Page 28, first paragraph - specify the age groups in the main text. Consider including the specific mean connectivities of the youngest and oldest groups as an example comparison.***

A18: We now added this information in page 23 of the new manuscript:

“The results revealed that the mean of the methylation connectivity within the atypical POE module 3 progressively increased with age (mean of the absolute correlation: 0.163 (0.156-0.171) for the 18-27y group and 0.315 (0.305-0.326) for the 66-94y group.”

***Q19: Acronyms, general comment - define acronyms before their first use to aide unfamiliar readers. For example, write out the full GS:SFHS study name at its first mention in the Introduction (page 9, lines 1-2) and in the Population Samples subsection of the Methods (page 10). The study could also be fully spelled out in Figure 1. Also, "PCs" on page 27, as that will be its first use.***

A19: These have been corrected in the page 7 (first mention of GS:SFHS in the Introduction), page 20 (first mention of PC in the Results) and page 36 (mention of GS:SFHS in the Methods) in the new manuscript. In the new Figure 1, we now have added the logo of Generation Scotland.

***Reviewer #2: Gao, et al. present a very strong and comprehensive analysis of an important emerging topic, parent-of-origin effects in DNA methylation and the association of methylation at the CpG loci with various aging-related traits.***

We thank the reviewer for these encouraging comments. We now carefully address the reviewer’s comments as below.

***Q1: One issue with the data, as presented, is that the analysis is of methylation sites previously demonstrated to have differential methylation profiles depending on the parent-of-origin. Thus, the analysis itself does not truly speak to differences in long-term health based on the maternal vs paternal origin of methylation changes, but rather informs on the extent to which such POE-dependent sites influence long-term health and disease. This isn't a major problem, but the title and abstract could make the true nature of the analysis and results clearer.***

A1: we apologize for the confusion. We have now changed the title as “Phenome-wide analyses identify an association between the parent-of-origin effects dependent methylome and the rate of aging in humans”.

***Some further concerns could be addressed to strengthen what is, overall, a very strong manuscript.***

***Introduction:***

***Q2: "Biological aging explained by DNA sequences, however, only accounts for influences from predisposing and unchangeable risk factors". This is specific to "heritable/constitutive DNA variation", as somatic DNA changes obviously influence biological aging. Mosaic loss of entire chromosomes are one such example outside the context of neoplastic disease.***

A2: we have modified the description as (page 4 in the new manuscript):

“The proportion of biological aging explained by the heritable DNA sequence variation, however, only accounts for the influences from predisposing and unchangeable risk factors.”

***Q3: "When it comes to early effects, a few studies reported association between early exposures and age acceleration in newborns and children, but not in adults (not tested)(13, 14)." Although an exhaustive list of such studies is unnecessary, it may be valuable to cite somewhere the emerging analyses of age-acceleration in newborns with Trisomy 21 (eg, PMID: 35661546).***

A3: we now have modified the description and referenced the mentioned study in page 4 in the new manuscript:

“When it comes to early effects, a few studies reported the associations of early exposures such as prenatal air pollution and early developmental event such as trisomy 21 with age acceleration in newborns and children(13, 14).”

***Q4: "To date, only one human study reported the association between methylation levels of POE-influenced genes and the change of brain structures overtime". "Overtime" should be two words.***

A4: we apologize for that. It is now corrected in page 6 in the new manuscript.

***Methods:***

***Q5: "The list of 984 POE-CpGs (Natypical=398, Ntypical=586) was extracted from a previous study(18)." This study was from these authors apparently using the same samples as the current Discovery set. There's nothing intrinsically wrong with such a design, but it should be made clear that the datasets nearly entirely overlap and acknowledge any caveats in reproducibility that this might entail.***

A5: We have added the following descriptions at the beginning of the Results section (page 9 of the new manuscript):

“Those CpGs displayed imbalanced methylation similarity between mother-offspring, father-offspring and siblings (Figure 1), and were identified using overlapping samples with the current discovery dataset (2).”

And the limitation section in discussion (page 34-35 of the new manuscript):

“First, the associations we reported were discovered and replicated in a Scottish population. The discovery dataset in this study used overlapping samples with the study that reported the imbalanced methylation features of POE-CpGs (2). Future studies are needed to replicate our findings in other populations.”

***Results:***

***A6: "The phenotypic categories contributed the largest number of associations were parental smoking exposure, lifestyle, intelligence and aging (Figure 3a)." "Contributed" should be "contributing".***

A6: we apologize for that. It is now corrected in page 10 of the new manuscript.

***Q7: "Conditional analyses suggested that the associations with parental smoking were mainly driven by maternal smoking". Is it possible to do these as purely stratified analyses? Or was paternal smoking data too sparse?***

A7: We understand the reviewer suggests testing of maternal smoking effects in samples with/without paternal smoking. This could be an interesting analysis, but with limited power in the data we have, as only a small proportion of samples in GS:SFHS contain records of parental smoking. In our datasets, only a small proportion of samples have linked data with birth cohorts, which are the major sources for us to collect information of paternal smoking. The data is indeed sparse: in all GS:SFHS samples (discovery+replication), there are only 1021 and 1883 samples have records for the maternal and paternal smoking status (ever smoke or not), respectively, 618 samples have information for both maternal and parental smoking status. There are only 272 offspring of the non-smoker fathers. For these, we think the conditional analyses would be better powered to test for the maternal/paternal smoking effect, and the results clearly pointed to a much stronger maternal smoking effect.

***Q8: "Conditional analyses indicated that the multiple associations of these POE-CpGs were not driven by the socioeconomic status (measured as SIMD)(Table s8)." This should be expanded using methylation as outcome and modeling all phenotypes together to assess whether smoking (or even IQ) is mediating the other phenotypic associations.***

A8: As described in the Methods (page 38,39, 41 in the new manuscript), all association tests, except the tests for the smoking status itself, have already accounted for the smoking effects by pre-correcting for the two smoking variables (smoking status and pack years)(the MOA model for the single-CpG association tests and the module-based association tests) or jointly fitting the two smoking variables as covariates (the CpG-outcome model for the single-CpG association tests). We now have also added a description in the Discussion (page 31) to emphasise this:

“Here, we uncovered its additional associations with education, socioeconomic status and DNAmTL acceleration (all of these associations are identified after smoking effects were accounted for).”

***Discussion:***

***A9: Some discussion of the potential for genetic confounding to play a role in amplifying results seems merited. How much is maternal/paternal IQ influencing parental behavior and child exposures, and playing out here as methylation-associations with subject intelligence (when just mediated by classical genetic SNP-based associations)?***

Q9: A very good point! We now have added the following sentences in the discussion in page 35 of the new manuscript:

“Fifth, POE-CpGs’ associations in the offspring with heritable traits such as intelligence could tag the genetic-mediated environmental effects from parental behaviors, which have a genetic component relating to the parents’ intelligence. Future POE studies accounting for the genetic nurture will be of great interest to disentangle these effects.”

**References**

1.	Plasschaert RN, Bartolomei MS. Genomic imprinting in development, growth, behavior and stem cells. Development. 2014;141(9):1805-13.

2.	Zeng Y, Amador C, Xia C, Marioni R, Sproul D, Walker RM, et al. Parent of origin genetic effects on methylation in humans are common and influence complex trait variation. Nature communications. 2019;10(1):1383.

3.	Benjamini Y, Yekutieli DJAos. The control of the false discovery rate in multiple testing under dependency. 2001:1165-88.

4.	Conesa A, Madrigal P, Tarazona S, Gomez-Cabrero D, Cervera A, McPherson A, et al. A survey of best practices for RNA-seq data analysis. 2016;17(1):1-19.

5.	Zhang F, Chen W, Zhu Z, Zhang Q, Nabais MF, Qi T, et al. OSCA: a tool for omic-data-based complex trait analysis. Genome biology. 2019;20(1):107.

6.	Lawson HA, Cheverud JM, Wolf JB. Genomic imprinting and parent-of-origin effects on complex traits. Nature Reviews Genetics. 2013;14(9):608-17.

7.	Lawson HA, Cheverud JM, Wolf JB. Genomic imprinting and parent-of-origin effects on complex traits. Nature reviews Genetics. 2013;14(9):609-17.

8.	Monk D, Mackay DJG, Eggermann T, Maher ER, Riccio A. Genomic imprinting disorders: lessons on how genome, epigenome and environment interact. Nature reviews Genetics. 2019.

9.	Cortessis VK, Azadian M, Buxbaum J, Sanogo F, Song AY, Sriprasert I, et al. Comprehensive meta-analysis reveals association between multiple imprinting disorders and conception by assisted reproductive technology. 2018;35(6):943-52.

10.	Taniguchi K, Kawai T, Hata K. Placental Development and Nutritional Environment. Adv Exp Med Biol. 2018;1012:63-73.

11.	Carpenter BL, Zhou W, Madaj Z, DeWitt AK, Ross JP, Gronbaek K, et al. Mother-child transmission of epigenetic information by tunable polymorphic imprinting. Proceedings of the National Academy of Sciences of the United States of America. 2018;115(51):E11970-E7.

12.	Derakhshan M, Kessler NJ, Ishida M, Demetriou C, Brucato N, Moore Gudrun E, et al. Tissue- and ethnicity-independent hypervariable DNA methylation states show evidence of establishment in the early human embryo. Nucleic acids research. 2022.

13.	Martens DS, Cox B, Janssen BG, Clemente DB, Gasparrini A, Vanpoucke C, et al. Prenatal air pollution and newborns' predisposition to accelerated biological aging. 2017;171(12):1160-7.

14.	Xu K, Li S, Muskens IS, Elliott N, Myint SS, Pandey P, et al. Accelerated epigenetic aging in newborns with Down syndrome. Aging Cell. 2022;21(7):e13652.

**Second round of review**

**Reviewer 1**

The authors have addressed our concerns in the revised version of the manuscript

**Reviewer 2**

I am very pleased with the authors' efforts to respond to a long list of reviewer comments. They have improved their paper considerably and I believe that it will be an impactful publication.
